# Targetron-Assisted Delivery of Exogenous DNA Sequences into *Pseudomonas putida* through CRISPR-Aided Counterselection

**DOI:** 10.1021/acssynbio.1c00199

**Published:** 2021-10-02

**Authors:** Elena Velázquez, Yamal Al-Ramahi, Jonathan Tellechea-Luzardo, Natalio Krasnogor, Víctor de Lorenzo

**Affiliations:** † Systems and Synthetic Biology Department, Centro Nacional de Biotecnología (CNB-CSIC), Campus de Cantoblanco, Madrid 28049, Spain; ‡ Interdisciplinary Computing and Complex Biosystems (ICOS) Research Group, Newcastle University, Newcastle Upon Tyne NE4 5TG, U.K.

**Keywords:** Pseudomonas putida, targetron, genome editing, CRISPR/Cas9, barcode, orthogonal DNA

## Abstract

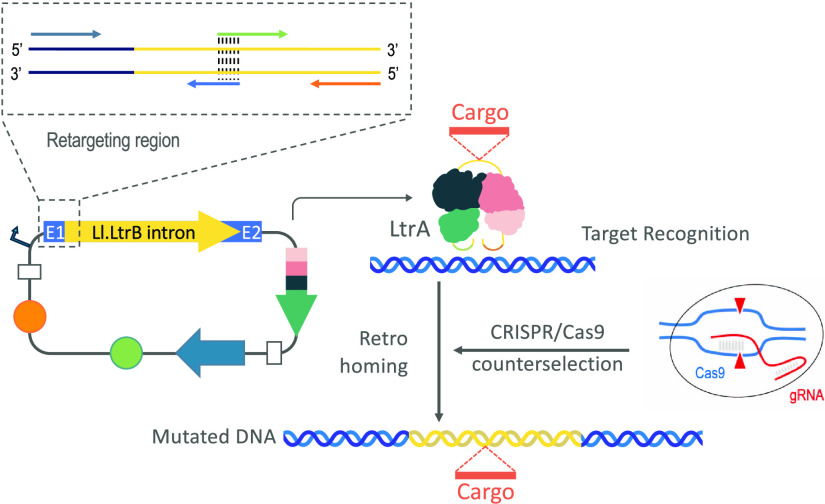

Genome editing methods based on group II introns (known as targetron technology) have long been used as a gene knockout strategy in a wide range of organisms, in a fashion independent of homologous recombination. Yet, their utility as delivery systems has typically been suboptimal due to the reduced efficiency of insertion when carrying exogenous sequences. We show that this limitation can be tackled and targetrons can be adapted as a general tool in Gram-negative bacteria. To this end, a set of broad-host-range standardized vectors were designed for the conditional expression of the Ll.LtrB intron. After establishing the correct functionality of these plasmids in *Escherichia coli* and *Pseudomonas putida*, we created a library of Ll.LtrB variants carrying cargo DNA sequences of different lengths, to benchmark the capacity of intron-mediated delivery in these bacteria. Next, we combined CRISPR/Cas9-facilitated counterselection to increase the chances of finding genomic sites inserted with the thereby engineered introns. With these novel tools, we were able to insert exogenous sequences of up to 600 bp at specific genomic locations in wild-type *P. putida* KT2440 and its Δ*recA* derivative. Finally, we applied this technology to successfully tag *P. putida* with an orthogonal short sequence barcode that acts as a unique identifier for tracking this microorganism in biotechnological settings. These results show the value of the targetron approach for the unrestricted delivery of small DNA fragments to precise locations in the genomes of Gram-negative bacteria, which will be useful for a suite of genome editing endeavors.

*Pseudomonas putida* is a soil bacterium and plant root colonizer that has emerged as one of the species with the highest potential as a synthetic biology chassis for industrial and environmental applications.^1,2^ Qualities of interest include the lack of pathogenicity,^3^ its high tolerance to oxidative stress^4,5^ (a most desirable trait in processes such as biofuel production^6^), diverse and powerful capabilities for catabolizing aromatic compounds,^7−9^ and ease of genetic and genomic manipulations.^10−13^ In particular, a suite of molecular tools have become available for the deletion and insertion of exogenous sequences in the genome of this bacterium, directed either to random locations (e.g., with transposon vectors^14,15^) or to specific genomic loci through recombineering^16^ or homologous recombination^13^ (reviewed in ref (17)). In this last and most widely used case, recombination efficacies vary considerably among different bacterial groups and even strains of the same species. For example, editing in the archetypal *P. putida* KT2440 is particularly suboptimal in *recA-*dependent processes.

Group II introns could be an alternative editing technique in cases with low recombination-based editing efficiency, as they work in a *recA*-independent fashion. Group II introns are a type of retroelement with the capacity to self-splice from an mRNA and insert stably into specific DNA loci (a process known as retrohoming^18,19^). Their conserved tertiary structure and a protein encoded within the retroelement (IEP or intron-encoded protein) are key components for the splicing and recognition of the target DNA.^20,21^ After translation, the IEP binds specifically to the catalytic RNA and first assists in the splicing of the intron from the containing exons. Afterward, both the IEP and the spliced intron stay together, forming a ribonucleoprotein (RNP) that carries out the recognition, reverse splicing, and retrotranscription of the intronic RNA into a new DNA site.^22^ In the past, several of these introns have been engineered to recognize and insert into specific genes different from their native retrohoming sites, giving rise to the knockout system named targetron.^23^ This platform is founded on the Ll.LtrB group II intron from *Lactococcus lactis* since it is the most studied case and it was proven to work in a wide range of bacterial genera, from *Clostridium*(24) or *Bacillus*(25) to the well-characterized species *Escherichia coli*.^23^ Later, targetrons were assayed for the delivery of specific cargo sequences into designated loci.^26−28^ Group II introns are promising tools to this end as they give rise to stable integrations.^26,28^ Another useful feature is that they are broad-host-range actors that can work in a great variety of organisms.^24,25,27,29,30^ Moreover, they can be redirected to virtually any desired genomic location with high specificity.^23,31^ Finally, as already mentioned, they can be an alternative to homologous recombination-based techniques, as they function independently of *recA*, which is a considerable advantage compared to other editing systems.^32,33^

Unfortunately, the targetron system also has some shortcomings. First, targetrons can be modified to recognize new sequences, but integration efficiency greatly changes depending on the new target site. Algorithms have been developed to identify the best retargeting options in a given sequence for Ll.LtrB^23,24^ and also for other group II introns.^31^ These algorithms retrieve a list of integration loci near a target site, ordered by a predicted score, and they also design primers for the modification of the recognition sequences inside the intron. However, as a result of their probabilistic nature, these predictions are not always reliable. Second, while cargo sequences can be inserted inside of group II introns, e.g., in the IVb domain,^27^ their addition lowers the efficiency of splicing and retrohoming. Therefore, despite the good characteristics of the platform, the application of group II introns as genome editing tools has not been widespread. Recently, some efforts to overcome these drawbacks were made when CRISPR/Cas9 technology was merged with targetrons to increase the recovery efficiency of successful mutants in *E. coli*.^33^ In this case, successful targetron editing events are selected by exploiting the CRISPR/Cas9 machinery^34^ as a way of counterselecting against nonmutated, wild-type sequences.^35,36^ By designing gRNAs that recognize the target WT DNA and cleaving the cognate unedited genome site with Cas9/gRNA, the chance of finding correctly edited mutants increases greatly. However, the value of merging Ll.LtrB insertions with Cas9/gRNA counterselection and the tolerance of the system to exogenous cargos of different sizes have not been tackled thus far in other species. The design of a broad-host-range platform including all of the components of the system is thus required.

The work below describes a set of standardized plasmids expressing the Ll.LtrB intron under the control of different promoters (with IPTG or cyclohexanone induction^37^), origins of replication, and antibiotic resistance genes that all work in a suite of Gram-negative bacteria. To characterize the performance of the new expression plasmids, we engineered them to insert Ll.LtrB-carrying cargos of increasing sizes into specific genomic locations of *E. coli* and *P. putida*, in a fashion that can be easily counterselected for with the gRNA/Cas9 system mentioned above.^36^ Furthermore, we show that the performance of the platform is independent of *recA*. Finally, we adopted this technology to successfully label *P. putida* KT2440 with a specific synthetic barcode for the identification and tracking of this strain in biotechnological applications. By introducing these barcodes, a physical link is created between the engineered organism and a digital twin.^39^ As explained below, this enables a version control system for microbial strains where all important information can be archived and consulted whenever necessary.

## Results and Discussion

### Engineering Broad-Host Expression of Intron Ll.LtrB

The Ll.LtrB intron and commercial targetron technology (Supplementary Figure S1A) have been exploited to work in diverse bacteria,^23,24,27,29,30,38^ but the vectors involved had to be modified in each case. In an attempt to make the same methodology more accessible and generalizable, we first set out to merge the key parts and properties of the Ll.LtrB system with standardized SEVA (Standard European Vector Architecture)^12,39,40^ plasmids.

SEVA vectors are composed of interchangeable modules including broad-host-range origins of replication, antibiotic resistance genes, and a wide set of expression systems and reporter genes. To bring the SEVA standard to targetron technology, we constructed a collection of plasmids expressing the Ll.LtrB intron under the control of alternative expression systems and selectable markers that can be adapted for multiple purposes.

**Figure 1:**
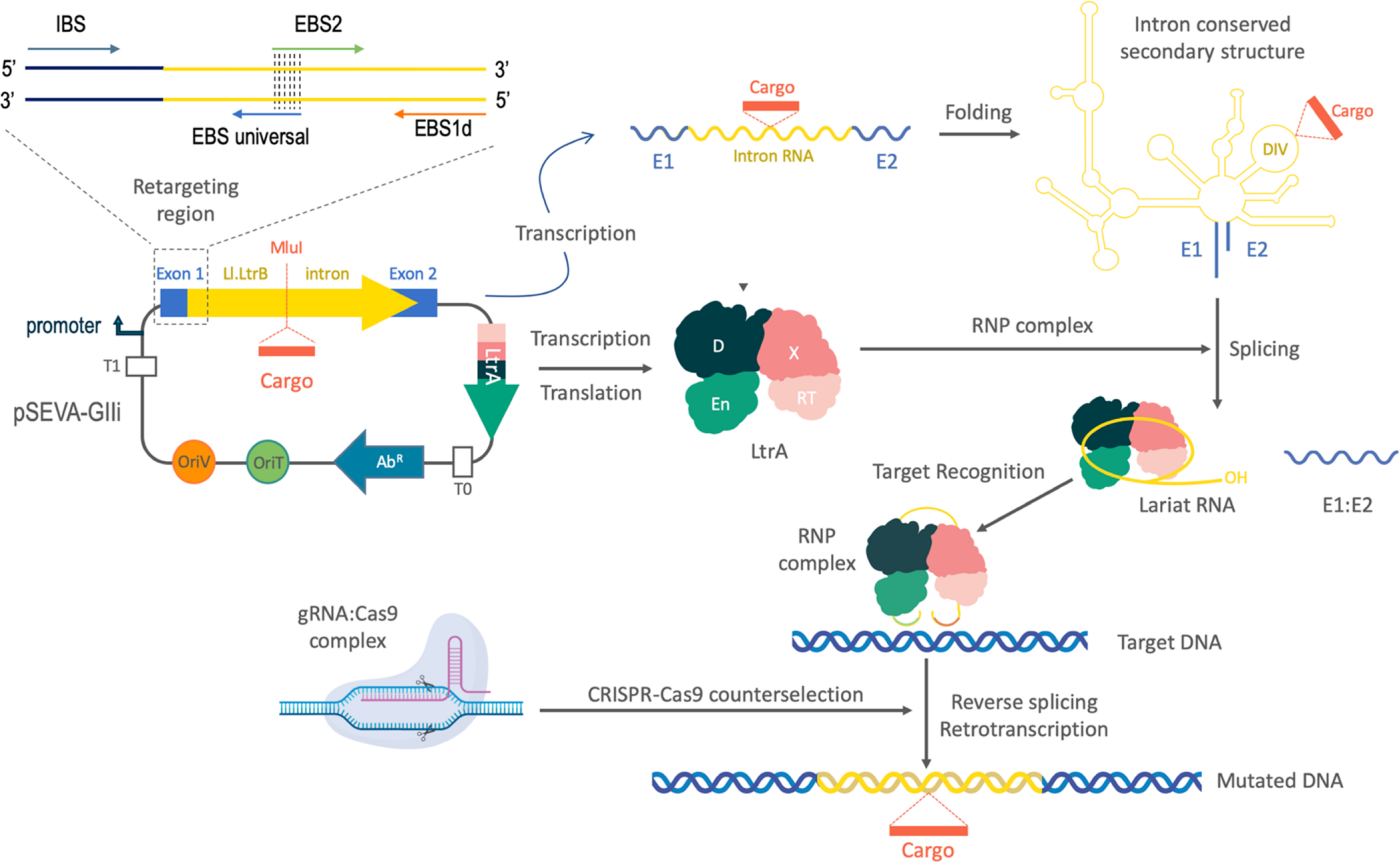
Diagram of targetrons and CRISPR/Cas9-mediated counterselection of insertions. The figure shows the technology developed in this article. Plasmid pSEVA-GIIi expresses the Ll.LtrB group II intron (empty or with cargo sequences cloned in the *Mlu*I site) and its IEP (LtrA) in the same transcriptional unit from the upstream promoter. The transcriptional unit is led by a retargeting region at 5′ (including exon 1 and the 5′ sequence of the Ll.LtrB intron), where three short sequences retrieved from the target gene (IBS, EBS1d, and EBS2) were engineered at given sites of the predicted transcript to secure its proper folding and retargeting (location of diagnostic primers is indicated). After transcription, the intronic RNA folds into a very conserved secondary structure and associates with LtrA to perform the splicing process from the exons. A lariat RNA is generated that remains attached to LtrA, forming a ribonucleoprotein (RNP) complex. This RNP scans DNA molecules until it finds the target site for retrohoming. Reverse splicing links covalently the intronic RNA to the sense strand of the DNA molecule, and the endonuclease (En) domain present in LtrA cleaves the antisense strand. Afterward, the retrotranscriptase (RT) domain of LtrA reverse transcribes the intronic RNA into DNA. The complete integration and synthesis of the cDNA is driven by repair mechanisms without the involvement of recombination. The incorporation of Cas9 complexes with gRNAs that recognize the insertion locus of Ll.LtrB causes the elimination of nonedited cells, as only those which incorporated the group II intron at the correct locus can survive (counterselection). IBS1: intron-binding site 1; EBS1d: exon-binding site 1-δ; EBS2: exon-binding site 2.

Figure 1 shows the whole experimental pipeline for the site-specific insertion of cargo in target genomic sites enabled by the pSEVA-Glli vector series. To benchmark the workflow, plasmid pSEVA421-GIIi (Km) was assembled (Supplementary Figure S1B). This construct is a low copy number (RK2 origin of replication) streptomycin/spectinomycin resistance plasmid carrying adjacent polycistronic Ll.LtrB intron and LtrA (Ll.LtrB IEP) sequences under the control of a T7 promoter. The Ll.LtrB segment of pSEVA421-GIIi (Km) has a retrotransposition-activation selectable marker (RAM) cargo composed of kanamycin resistance (Km^R^) gene interrupted by a group I intron (black square inside the Km^R^ gene in the Supplementary Figure S1A,B,H) in domain IVb, which has been previously shown to increase the likelihood of finding retrohomed mutants.^41^ The construct is arranged in a way that only if the Ll.LtrB intron is spliced and inserted, the group I intron is excised and the Km^R^ gene is reconstituted. Therefore, selection in Km plates facilitates the identification of insertion mutants. Finally, the Ll.LtrB borne by pSEVA421-GIIi (Km) is retargeted to insert into position 1063 of the *lacZ* gene of *E. coli* in antisense orientation. The expectation is that upon correct Ll.LtrB retrohoming and insertion into a target genomic sequence (e.g., *lacZ*), the group I intron will be lost, the Km^R^ restored, and the β-galactosidase gene interrupted, allowing for easy screening of the site-specific cargo insertion.

**Figure 2:**
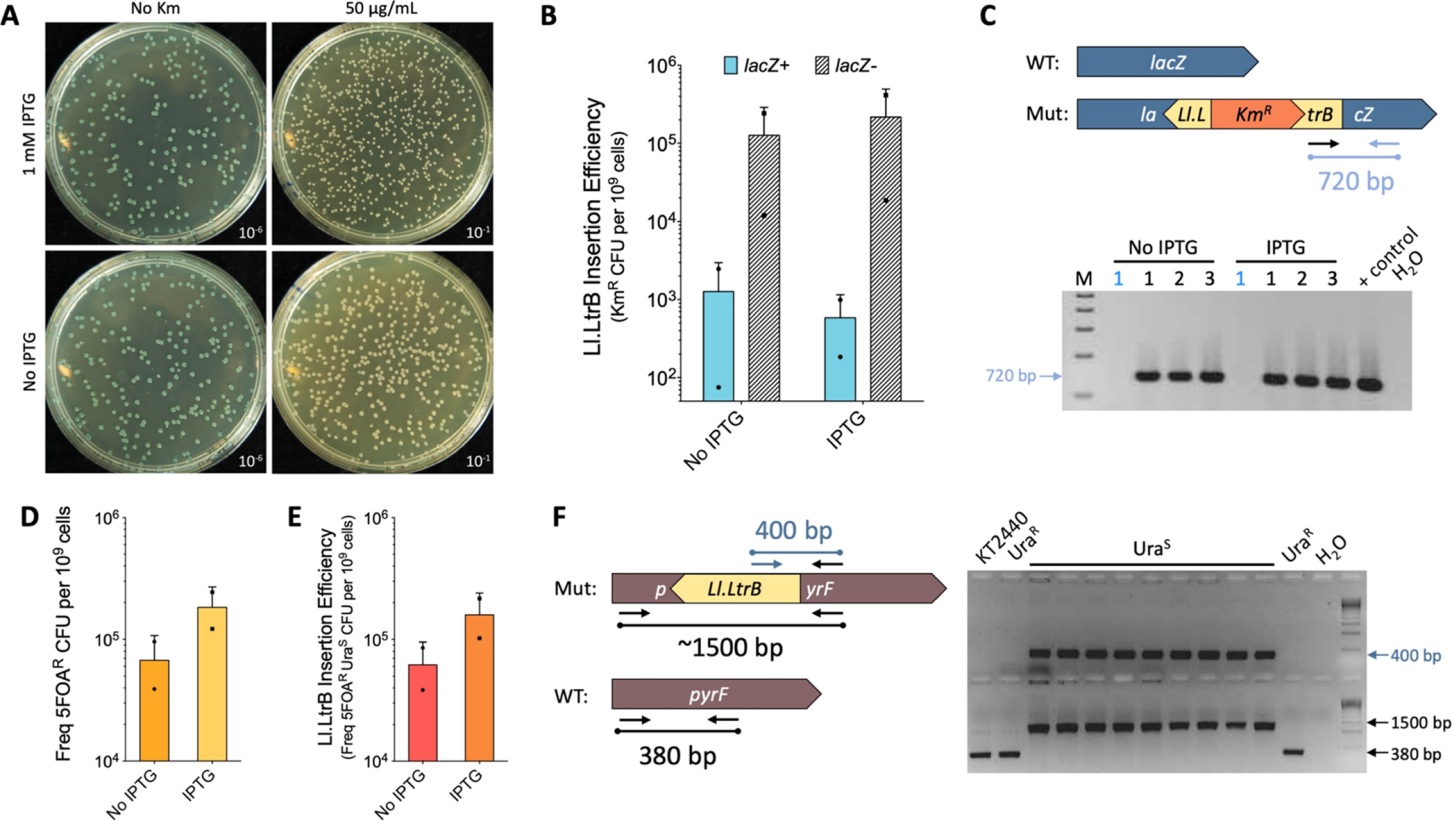
SEVA plasmids encoding the Ll.LtrB group II intron and T7 RNAP work in *E. coli* BL21DE3 and *P. putida* KT2440. (A) Delivery of the Ll.LtrB intron from plasmid pSEVA421-GIIi (Km) in *E. coli* BL21DE3. The Ll.LtrB intron was retargeted to insert in the antisense orientation into the locus 1063 of the *lacZ* gene so that insertions would disrupt this gene, giving rise to white colonies in the presence of X-gal. Since a RAM is placed inside Ll.LtrB, kanamycin resistance was used as a way to select for intron insertion mutants (plates to the right). (B) Graph shows the number of Km^R^ CFU normalized to 10^9^ viable cells and classified according to the displayed phenotype in the presence of X-gal (blue colonies: *lacZ*^+^, blue bars; white colonies: *lacZ*^–^ (disrupted), white hatched bars) and, also, according to the presence or absence of IPTG induction. (C) Representative colony PCR reactions to determine the correct insertion of Ll.LtrB inside the *lacZ* gene. Only if Ll.LtrB retrohomes, a PCR amplicon of 720 bp is generated. Blue numbers correspond to blue colonies, and black numbers correspond to white colonies used as the template material for each reaction. (D–F) Delivery of Ll.LtrB from plasmid pSEVA421-GIIi-pyrF and with the help of pSEVA131-T7RNAP in *P. putida* KT2440. (D) Bar plot showing the frequency of 5FOA^R^ CFU normalized to 10^9^ viable cells after the insertion assay. CFU are classified according to the addition or absence of IPTG during the incubation period. (E) Genuine efficiency of the insertion of Ll.LtrB. The proportion of uracil auxotrophs detected from the 5FOA^R^ population was used as a ratio to determine the abundance of Ll.LtrB insertions in the population. (F) 5FOA counterselection was used to isolate insertion mutants that were not able to grow without uracil supplemented to plates. Colonies resistant to 5FOA but that were able to grow without uracil were used as negative controls of insertion. Two different PCR reactions are shown: (top gel) one primer annealed inside Ll.LtrB and the second annealed in the *pyrF* gene so that an amplicon could be only generated after intron insertion. (Bottom gel) two primers flanking the insertion locus were used so that two amplicons could be generated. The smallest fragment (380 bp) corresponds to the WT sequence, and the biggest fragment (∼1500 bp) corresponds to the insertion. The same colonies were tested in both PCR reactions. All bar graphs show the mean values (bars), standard deviation (lines), and single values (dots) of two biological replicates. WT: wild-type; Mut: insertion mutant; + control: reaction with a colony with successful insertion from a previous experiment used as a template; H_2_O: control PCR with no template material; Ura^R^: colonies growing in media without uracil (no auxotrophs); Ura^S^: colonies not growing in media without uracil (auxotrophs).

With this expectation, pSEVA421-GIIi (Km) was tested in *E. coli* BL21DE3 (Figure 2A–C). This strain was chosen as it provides the T7 RNAP^42^ that is necessary for the expression of the whole plasmid insert. As the cloned group II intron was retargeted to insert into the *lacZ* gene, blue/white screening in X-gal plates was used to visually quantify the efficiency of the insertion process. A sample of the blue/white screenings of such insertion experiments with pSEVA421-GIIi (Km) is shown in Figure 2. The screens indicated that the Ll.LtrB intron was retrohoming to the selected locus inside the *lacZ* gene with good efficiency (Figure 2A). When Km was added to plates (Figure 2A, right plates), the number of white colonies was boosted in comparison to the plates with no selection (Figure 2A, left plates). Note that the induction of the T7 RNAP in the host strain *E. coli* BL21DE3 with IPTG did not make much difference in the frequencies of the process, which is likely due to the leakiness of the *lacUV5* promoter that drives the expression of the polymerase.^43,44^ In any case, the efficiency of the insertion of Ll.LtrB from pSEVA421-GIIi (Km) was calculated to be ca. 2.1 × 10^–4^ (in the +IPTG conditions).

We also spotted a low number of Km^R^ LacZ^+^ colonies (∼1% of total Km^R^ clones) that was indicative of illegitimate off-target insertions, perhaps due to retrotransposition instead of retrohoming.^45−47^ However, the frequency of such events was >200-fold less than those with the correct insertion phenotype (Figure 2B). Finally, colony PCR of white and blue colonies was performed to check the precise insertion of the group II intron into the *lacZ* gene and the correlation with the observed phenotype (Figure 2C). To this end, a total of 10 white colonies of the Km-containing plates were screened from each condition (induction versus noninduction), and all of them had incorporated Ll.LtrB in the expected site. Three Km^R^ LacZ^+^ colonies from the same plates were also used as negative controls; all three failed to amplify, indicating no insertion of Ll.LtrB inside the *lacZ* gene.

### Ll.LtrB Intron Retrohomes in *P. putida* KT2440

After testing the efficiency of the SEVA-based constructs described above in *E. coli*, we next inspected the activity of the same Ll.LtrB in *P. putida*. For this, we first constructed a compatible pSEVA vector for the heterologous expression of the T7 RNAP. The complete sequence of this ORF, along with the regulatory regions for IPTG-controlled expression (*lac*UV5 promoter along with a short 5′ region of the *lacZ* gene fused to the T7 RNAP ORF), was cloned into pSEVA131, yielding pSEVA131-T7RNAP (Supplementary Figure S1C). This plasmid bears an ampicillin resistance gene (Ap^R^), a pBBR1 origin of replication, which confers a medium copy number of plasmids, and is compatible with pSEVA421-GIIi (Km). The target gene of choice for benchmarking the method in *P. putida* was not *lacZ* (which is absent in this species) but *pyrF* (*PP1815*), the orthologue of the yeast *URA3* gene for orotidine-5′-phosphate decarboxylase (ODCase).^48^ The loss of this marker can be selected both negatively (*pyrF* mutants become auxotrophic for uracil) and positively (the same mutants are also resistant to 5-fluoroorotic acid, 5FOA^49^). Since such a double selection works well in *P. putida* KT2440,^50^ we adopted *pyrF* as a suitable candidate for the insertion of Ll.LtrB. For this, the Ll.LtrB was retargeted to insert into a specific locus inside the *pyrF* ORF, giving rise to pSEVA421-GIIi (Km)-pyrF. This plasmid is equivalent to the pSEVA421-GIIi (Km) used in *E. coli* (see above), but the retargeting region (Figure 1) was engineered to carry *pyrF* sequences instead of *lacZ* segments. A variant of this plasmid, which lacked the RAM used in the case of *E. coli* (see above), named pSEVA421-GIIi-pyrF (Supplementary Figure S1D), was also built to compare the efficacies of the different selection methods.

Both plasmids (i.e., with and without RAM) were transformed into *P. putida* KT2440 individually, in each case with pSEVA131-T7RNAP, and the insertion assay was performed as in *E. coli* above. After induction for 2 h with IPTG, the cultures were plated on a minimal medium supplemented with 5FOA and uracil to select for Ll.LtrB-retrohomed clones. While the frequency of 5FOA^R^ spontaneous mutants in *P. putida* KT2440^50^ is ∼0.8 × 10^–6^, the intron insertion procedure increased the ratio of 5FOA^R^ colonies to ∼10^–4^ (100-fold; Figure 2D). As in *E. coli*, IPTG addition increased only marginally the number of 5FOA^R^ mutants. A number of individual 5FOA^R^ clones were subsequently patched on plates with and without uracil to verify the *pyrF*-minus phenotype. Typically, 93.6% of 5FOA^R^ clones from the noninduced culture and 86.2% of that with IPTG turned out to be uracil auxotrophs, which allowed the calculation of *bona fide* frequencies of Ll.LtrB insertion (Figure 2E). In addition, PCR reactions (Figure 2F) authenticated the accuracy of Ll.LtrB incorporation at the expected site in *pyrF*.

These experiments certified the ability of Ll.LtrB to retrohome into a specific site of the genome of *P. putida* KT2440 in a fashion similar to other Gram-negative species.^38^ Intriguingly, selection of intron insertions based on RAM, which worked so well in *E. coli*(41) (see above) and other species,^51^ failed altogether to deliver valid clones in *P. putida*, regardless of whether the selection was made with Km or 5FOA. Such a malfunction (which has been reported before in *P. aeruginosa*(38)) cannot be blamed on the lack of processivity of RNAP (the RNA element is expressed from a T7 promoter^42,52,53^). It is instead possible that the excision of the group I intron in the RAM may depend on host-specific factors.

### Streamlining Ll.LtrB Expression and Activity in a Δ*recA* Derivative Strain of *P. putida* KT2440

**Figure 3:**
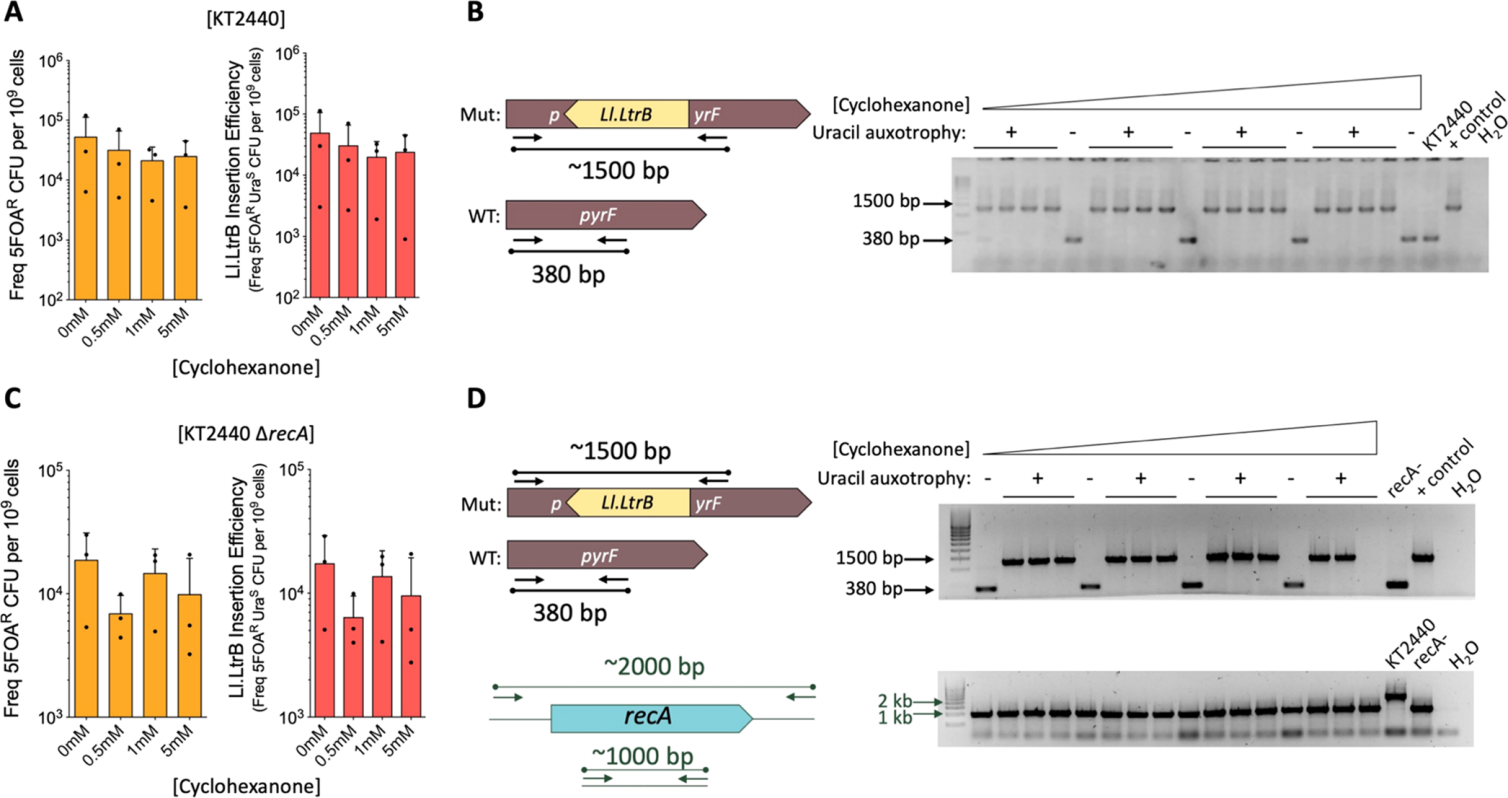
Performance of pSEVA2311-GIIi-pyrF in *P. putida* KT2440 and its Δ*recA* derivative strain. (A) pSEVA2311-GIIi-pyrF works in *P. putida* KT2440 to deliver the Ll.LtrB intron into the *pyrF* gene through 5FOA counterselection. Left panel: Bar plot showing the frequency of 5FOA^R^ CFUs normalized to 10^9^ viable cells of wild-type *P. putida* after the insertion assay resulting from induction with various cyclohexanone concentrations (0, 0.5, 1, and 5 mM). Right panel: Genuine efficiency of insertion of Ll.LtrB. The proportion of uracil auxotrophs detected from the 5FOA^R^ population was used as a reference to determine the abundance of Ll.LtrB insertions in each population. (B) Colony PCR reaction that used primers flanking the insertion locus was used to determine Ll.LtrB retrohoming in each concentration of cyclohexanone tested (from 0 mM in the left part of gel to 5 mM in the right part of gel). The smallest fragment (380 bp) corresponds to the wild-type sequence, while the biggest one (1500 bp) corresponds to the intron insertion in the correct location. (C, D) Functioning of pSEVA2311-GIIi-pyrF in *P. putida* KT2440 Δ*recA*. The same experiments and analyses were done to determine the frequencies and correctness of intron insertion in the recombination-deficient strain. The gel at the bottom of (D) shows a control PCR reaction to verify *recA* minus genotype of the tested cells. The frequency of 5FOA^R^ CFUs and the efficiency of insertion of Ll.LtrB in *P. putida* Δ*recA* were determined as before. All bar graphs show the mean values (bars), standard deviation (lines), and single values (dots) of at least three biological replicates. WT, wild-type; Mut, insertion mutant; KT2440, parental strain; + control: PCR of DNA from an intron-inserted colony (from a previous experiment) used as the template; H_2_O: control PCR with no template material.

While the data above demonstrate the functioning of the Ll.LtrB platform in *P. putida*, the two-vector system can be simplified to make the platform easier to use in practice. To simplify the system to a single plasmid, we took advantage of the standardized architecture of SEVA plasmids to transfer the DNA segment of pSEVA421-GIIi-pyrF bearing Ll.LtrB and LtrA into pSEVA2311.^37^ The result was the Km^R^ and pBBR1 *oriV* plasmid called pSEVA2311-GIIi-pyrF (Supplementary Figure S1E), in which the expression of Ll.LtrB is under the control of the broad-host-range cyclohexanone-inducible ChnR/P_ChnB_ promoter device.^37,54^ With this simplified tool in hand, we set out to compare the efficacy of Ll.LtrB insertion in the wild-type *P. putida* strain versus a recombination-deficient counterpart. To this end, *P. putida* KT2440 and an isogenic *recA* derivative were transformed with pSEVA2311-GIIi-pyrF, and the cultures of each transformant were grown and induced with 0, 0.5, 1, and 5 mM cyclohexanone. The samples were then plated on a minimal media with 5FOA and uracil to select for Ll.LtrB-inserted clones, and the colonies were analyzed as previously described. The results are shown in Figure 3. As in *E. coli* induced with IPTG, we found little difference in the number of 5FOA^R^ CFUs induced without or with varying cyclohexanone concentrations (Figure 3A,C, left plots). In fact, no or low induction levels gave rise to the highest frequency of 5FOA^R^ mutants in the Δ*recA* mutant (Figure 3C, left plot). As previously described, 5FOA^R^ colonies were patched on plates with and without uracil to search for authentic *pyrF* mutants, and the final frequency of 5FOA^R^/uracil auxotroph clones was calculated as an indication of the efficiency of insertion of Ll.LtrB for each cyclohexanone concentration (Figure 3A,C; right plots).

Finally, correct acquisition of the group II intron by *pyrF* was verified by means of colony PCR with primers flanking the site of insertion. Surprisingly, analysis of 5FOA^R^/uracil auxotroph colonies from both wild-type and Δ*recA* cultures (Figure 3B,D) indicated that cyclohexanone induction did not help to generate more valid insertions. We speculate that excessive induction of the engineered intron RNA along with the LtrA complex encoded in plasmid can be toxic and/or produce unexpected effects. In any case, identification of Ll.LtrB insertions in a *recA* defective strain validated the ability of group II introns to work in a recombinant-independent fashion in *P. putida*.^32,55^

### Merging Ll.LtrB Action with a CRISPR/Cas9-Mediated Counterselection System

Although the results above demonstrate the performance of group II intron insertion in *P. putida*, the measured frequencies of insertion were much too low to use the system as a practical genome editing tool when the pursued change is not selectable. On this basis, we sought to increase the insertion efficiency by combining the action of Ll.LtrB with the counterselection of unedited wild-type sequences with CRISPR/Cas9^33,36^—all formatted with tools following the SEVA standard. The existing broad-host-range system of reference for such a counterselection involves compatible plasmids pSEVA231-CRISPR (Km^R^, pBBR1 *oriV*) for cloning the spacer and pSEVA421-Cas9tr (Sm^R^, RK2 *oriV*) for the expression of Cas9 (Supplementary Table S2). This required the transfer of the DNA encoding the [ChnR/*P*_*ChnB*_ → Ll.LtrB/LtrA] device to a plasmid compatible with the other two. This process and the verification of the activity of the resulting intron delivery vectors are described in detail in the Supporting Information. The result of the exercise was pSEVA6511-GIIi-pyrF, as shown in Supplementary Figure S1F. This is a Gm^R^ plasmid with an RSF1010 *oriV*(12,56−58) compatible with pSEVA231-CRISPR and pSEVA421-Cas9tr and bearing a *pyrF*-retargeted Ll.LtrB and LtrA controlled by the ChnR/*P*_*ChnB*_ promoter.

**Figure 4:**
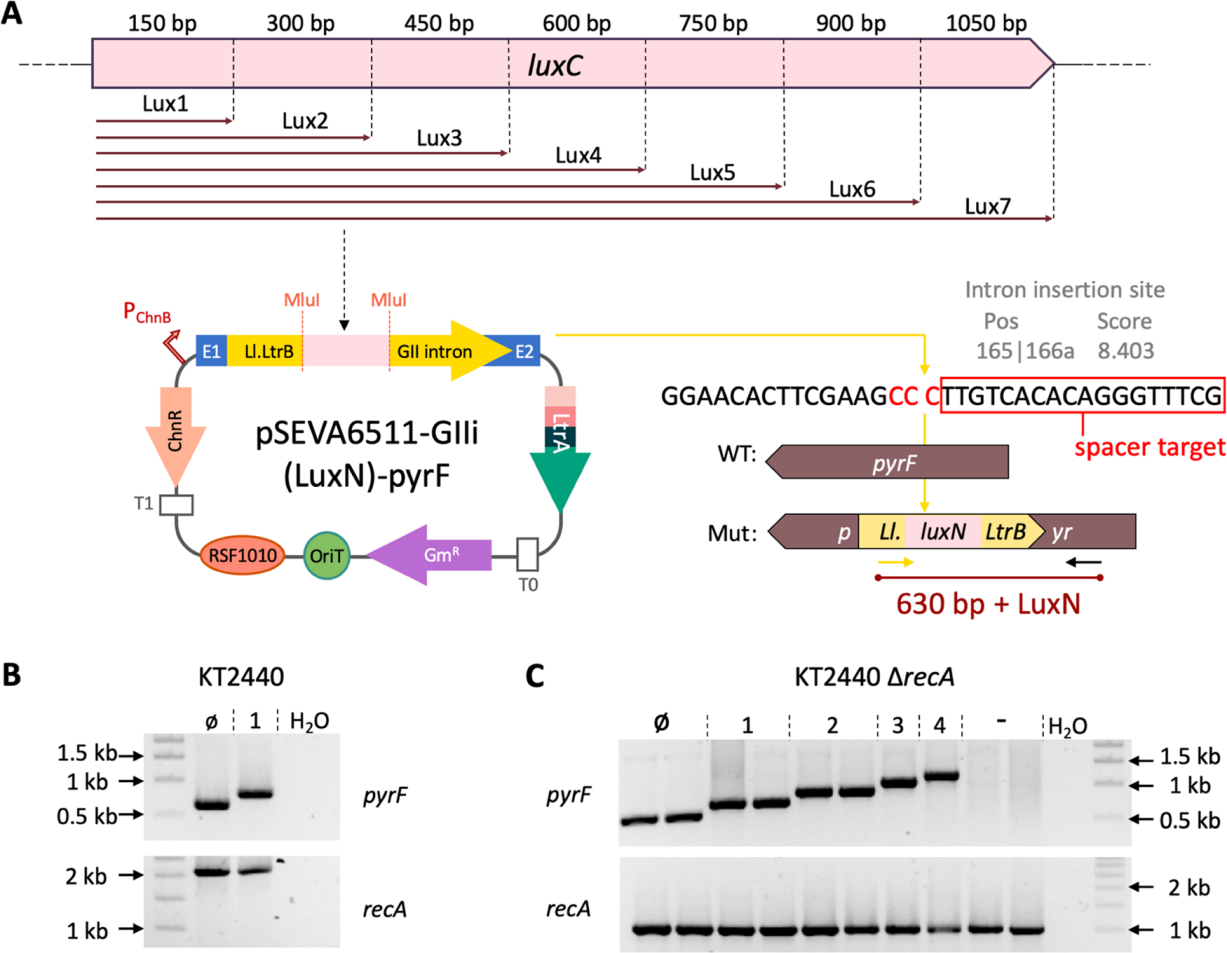
Assessing the size-restriction of intron-mediated delivery with CRISPR/Cas9 counterselection using *luxC* fragments as a cargo. (A) Schematic of the intron library generated with increasing fragment length as a cargo (from 150 up to 1050 bp) using as template the first gene of the *luxCDABEG* operon, *luxC*. The Ll.LtrB intron in pSEVA6511-GIIi (LuxN) is retargeted to insert between the nucleotides 165 and 166 of the *pyrF* ORF in the antisense orientation. Spacer pyrF1 recognizes the region after the insertion site (part of the recognition site is shown inside a red box). The complementary nucleotides to the PAM (5′GGG-3′) are highlighted in red and are disrupted upon intron insertion. (B) Ll.LtrB-mediated delivery of *luxC* fragments in *P. putida* KT2440 WT with CRISPR/Cas9 counterselection. Colony PCR reactions showing amplifications from colonies with Ll.LtrB::LuxØ and Ll.LtrB::Lux1 (top gel) and corresponding PCR reactions verifying the *recA*-plus phenotype (bottom gel). WT amplification for the *recA* gene is 2 kb long. (C) Ll.LtrB-mediated delivery of *luxC* fragments in *P. putida* KT2440 Δ*recA*. Colony PCR reactions showing amplifications from colonies with Ll.LtrB::LuxØ to Ll.LtrB::Lux4 (top gel) and corresponding PCR reaction verifying the *recA*—genotype (bottom gel). Deletion of the *recA* gene gives an amplification of 1 kb. WT: wild-type, Mut: insertion mutant, LuxN: cargos including from LuxØ to Lux7, Ø: Ll.LtrB with no cargo, 1: Ll.LtrB with Lux1 as the cargo, 2: Ll.LtrB with Lux2 as the cargo; 3: Ll.LtrB with Lux3 as the cargo, and 4: Ll.LtrB with Lux4 as the cargo. *P. putida* KT2440 Δ*recA* colonies with no inserted Ll.LtrB used as the negative control.

With this 3-plasmid platform in hand, we first set out to explore the limits in the size of fragments that can be delivered by the Ll.LtrB intron in *P. putida* KT2440 and its Δ*recA* derivative. Earlier work with other bacteria^26−28,33^ indicated that the length of the sequences inserted inside Ll.LtrB was critical for the splicing and retrohoming efficiency of the intron. In fact, in its native host, *L. lactis*, cargo sequences >1 kb abolished retrohoming.^27^ To determine the largest DNA fragment that Ll.LtrB was able to insert in both wild-type and Δ*recAP. putida*, we generated a library of pSEVA6511-GIIi-pyrF plasmid derivatives carrying fragments of increasing size out of the *luxC* gene (from 150 to 1050 bp; Figure 4A). The maximum insertion size without CRISPR counterselection was first assessed by performing the assay on plates with and without 5FOA-mediated counterselection, with insert sizes of 150 bp (Lux1), 600 bp (Lux4), 750 bp (Lux5), and 1050 bp (Lux7). In the case of wild-type *P. putida* KT2440, 5FOA counterselection delivered correct insertions up to Lux4 (600 bp) but not larger segments (Supplementary Figure S2 and Supplementary Table S3). In contrast, only the smallest DNA cargo (150 bp) could be counterselected with 5FOA in *P. putida* Δ*recA* (Supplementary Table S3).

**Figure 5:**
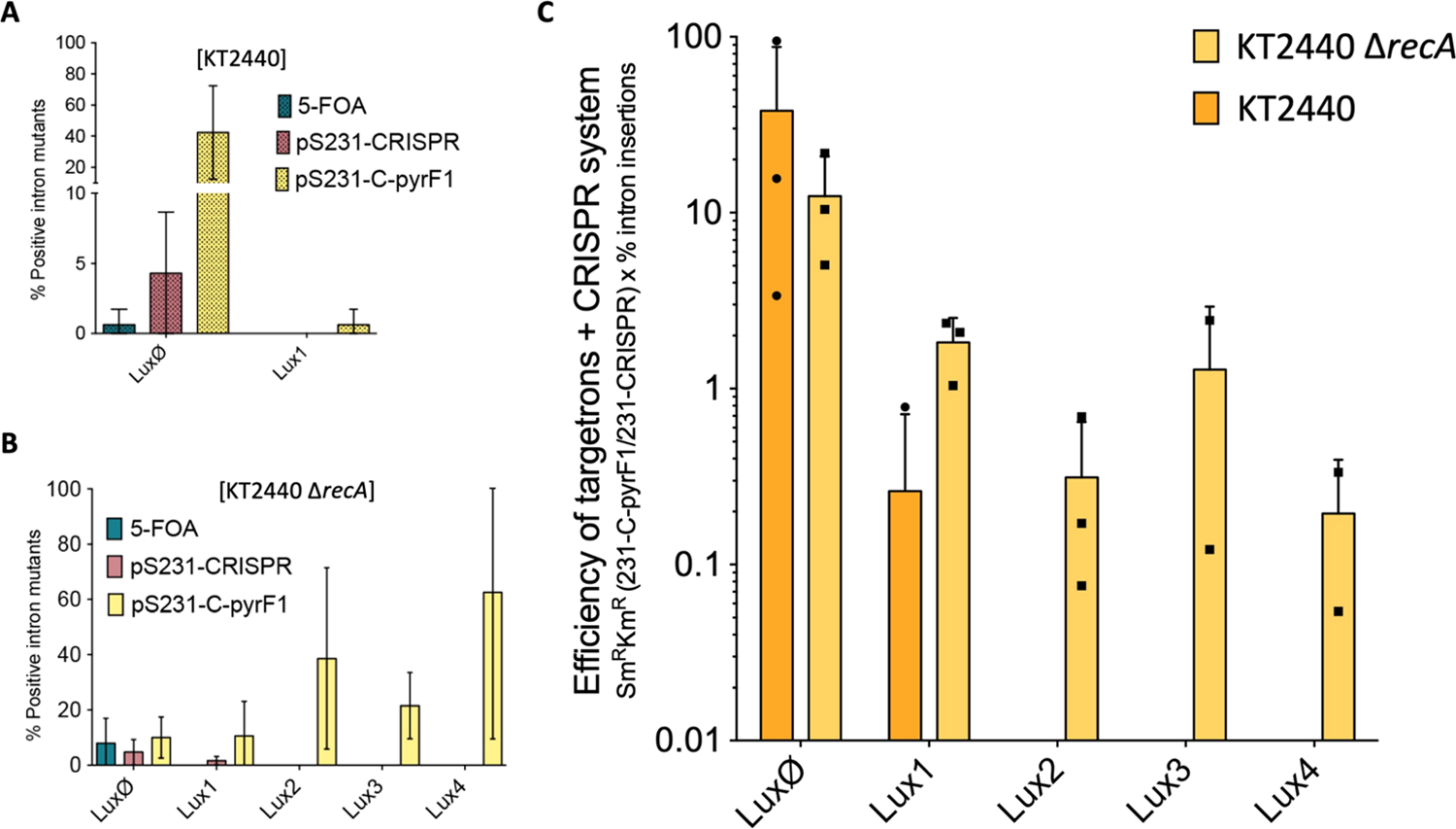
Intron insertion frequencies with CRISPR-Cas9 counterselection in wild-type and Δ*recAP. putida* KT2440 confirmed through PCR. (A) Intron insertion frequency of each cargo inserted in the genome of WT *P. putida* KT2440 by Ll.LtrB using 5FOA; no counterselection (control plasmid pSEVA231-CRISPR) or CRISPR/Cas9-mediated counterselection (pSEVA231-C-pyrF1). Insertion of a cargo larger than Lux1 was not detected. (B) Similarly, for *P. putida* KT2440 Δ*recA*, insertion of a cargo larger than Lux4 was not detected. (C) Combined efficiency of targetrons and CRISPR/Cas9 counterselection. The numbers of Sm^R^ Km^R^ CFU obtained after transforming either pSEVA231-CRISPR (control) or pSEVA231-C-pyrF1 were normalized to 10^9^ cells. The pSEVA231-CRISPR condition was set to 100%, and the percentage of cells with pSEVA231-C-pyrF1 was calculated accordingly. Finally, the ratio of positive Ll.LtrB insertions detected in the pSEVA231-C-pyrF1 condition was multiplied individually in each replicate. The mean (bars), single values (dots), and standard deviation (lines) of two or three replicates are shown. Ø: Ll.LtrB with no cargo; 1: Ll.LtrB with Lux1 as the cargo; 2: Ll.LtrB with Lux2 as the cargo; 3: Ll.LtrB with Lux3 as the cargo; and 4: Ll.LtrB with Lux4 as the cargo.

We next measured the efficacy as a function of insert size of selecting the same Ll.LtrB intron insertions by means of CRISPR/Cas9-based counterselection, which is expected to kill the wild-type population of nonmodified cells.^33,35,36,59^ For this, cells harboring both pSEVA421-Cas9tr^36^ and the corresponding pSEVA6511-GIIi (LuxN)-pyrF were grown overnight and then induced for 4 h with cyclohexanone. After this incubation time, an aliquot of these cells was plated in the presence of 5FOA to estimate the efficiency of Ll.LtrB insertions with this protocol and no CRISPR/Cas9 counterselection, as these cells contained no plasmid carrying a guide RNA. The rest of the cells were made competent and then separately transformed with either pSEVA231-CRISPR (negative control with no specific spacer) pSEVA231-C-pyrF1 (bearing a specific spacer whose PAM occurs in the wild-type insertion locus of Ll.LtrB::LuxN in *pyrF*(36)). The cells were then plated on LB media supplemented with Sm and Km to select for clones with both pSEVA421-Cas9tr and pSEVA231-CRISPR or pSEVA231-C-pyrF1. Our prediction was that if Ll.LtrB::LuxN retrohomes were expected, the genomic PAM sequence within *pyrF* necessary for Cas9 activation would be disrupted, and only these mutated cells would be able to survive (Figure 4A). By following this approach, mutated cells in both *P. putida* wild-type (Figures 4B and 5A,C) and Δ*recA* were isolated (Figures 4C and 5B,C). Interestingly, the wild-type strain did worse than the Δ*recA* variant in accepting longer DNA segments as intron cargos. Under the best conditions, as shown in Figure 4C, the upper limit in the insert size for the Δ*recA* strain was 600 bp (Lux4). As predicted, the frequency of correct insertions in either strain was boosted by pSEVA231-C-pyrF1 as compared to cells with the control plasmid pSEVA231-CRISPR (Supplementary Figure S3 and Supplementary Tables S4 and S5). In addition, counterselection allowed for the detection of longer inserts. In the absence of counterselection, no insertions of Ll.LtrB::Lux2–4 in the Δ*recA* strain could be identified (Figure 5B and Supplementary Table S5). Furthermore, Ll.LtrB::Lux1 retrohoming in the wild-type strain could be detected only when pSEVA231-C-pyrF1 had been transformed with the other CRISPR counterselection plasmids (Figure 5A,C).

To estimate the efficiency of the merged targetrons/CRISPR/Cas9 system, the numbers of Sm^R^ Km^R^ CFUs obtained after transforming either pSEVA231-CRISPR (control) or pSEVA231-C-pyrF1 to each strain were normalized to 10^9^ viable cells (Sm^R^ CFUs). This control condition was set to 100% and the percentage of pSEVA231-C-pyrF1 was calculated accordingly. Finally, the ratio of positive Ll.LtrB insertions detected through PCR for each case was calculated and multiplied accordingly in each replicate (Figure 5C). Taken together, the results indicated that the system, within the cargo size limits, enables the positive selection of Ll.LtrB integrations with their cargo by killing the population of nonmutated bacteria. The higher frequency of insertions detected in the Δ*recA* background is plausibly caused by the improved performance of CRISPR/Cas9 counterselection in recombination-deficient strains.^60^*RecA*-minus bacteria can neither use homologous recombination for repairing the double-stranded break catalyzed by Cas9 nor lose the CRISPR spacer cloned in pSEVA231-C-pyrF1, which is one of the main causes for escapers from the CRISPR/Cas9 counterselection to arise.^36^ It should be noted that the insertion frequencies may change depending on the genomic target of the intron^33^ and the sequence of the spacer in the CRISPR sequence.

Although the size of possible cargos that could be inserted in the *P. putida* genome was somewhat modest and the insertion frequencies decreased with their length, the results were in the range of those found in other bacterial types.^27,28^ This highlights the value of the Ll.LtrB platform for delivery of small fragments to specific genomic loci in *P. putida*; a real-world example use case is addressed in the next section.

### Application of Ll.LtrB for Barcoding Cells with Unique DNA Identifiers

A large number of biotechnological applications of *P. putida* could benefit from a targeted, stable genomic insertion of short synthetic and orthogonal DNA sequences (i.e., genetic barcodes) as unique identifiers of particular strains. These barcodes create a physical link between the tagged organism and a digital twin,^39^ which in turn enables a version control system for microbial strains where all important information can be archived and consulted.^61,62^ Given the broad host range and recombination-independent performance of the Ll.LtrB intron^27^ for directed insertion of small fragments of DNA, we evaluated its value for delivery of such barcodes/unique identifiers to the genomes of strains of interest. The barcodes of choice have a small size (148 bp, Supplementary Figure S4) and they are composed of a universal primer (25 nt), which is shared by all barcodes generated with CellRepo software,^61^ and a core sequence (123 nt). This is itself subdivided into three components: the barcode proper (96 nt), the synchronization sequence (9 nt), and the checksum (18 nt) component.^61^

The last two elements are incorporated as an error-correction mechanism.^61^ In this way, even if truncated or incorrect reads are retrieved, the CellRepo algorithm is still able to identify the barcode and its linked strain profile content.

**Figure 6:**
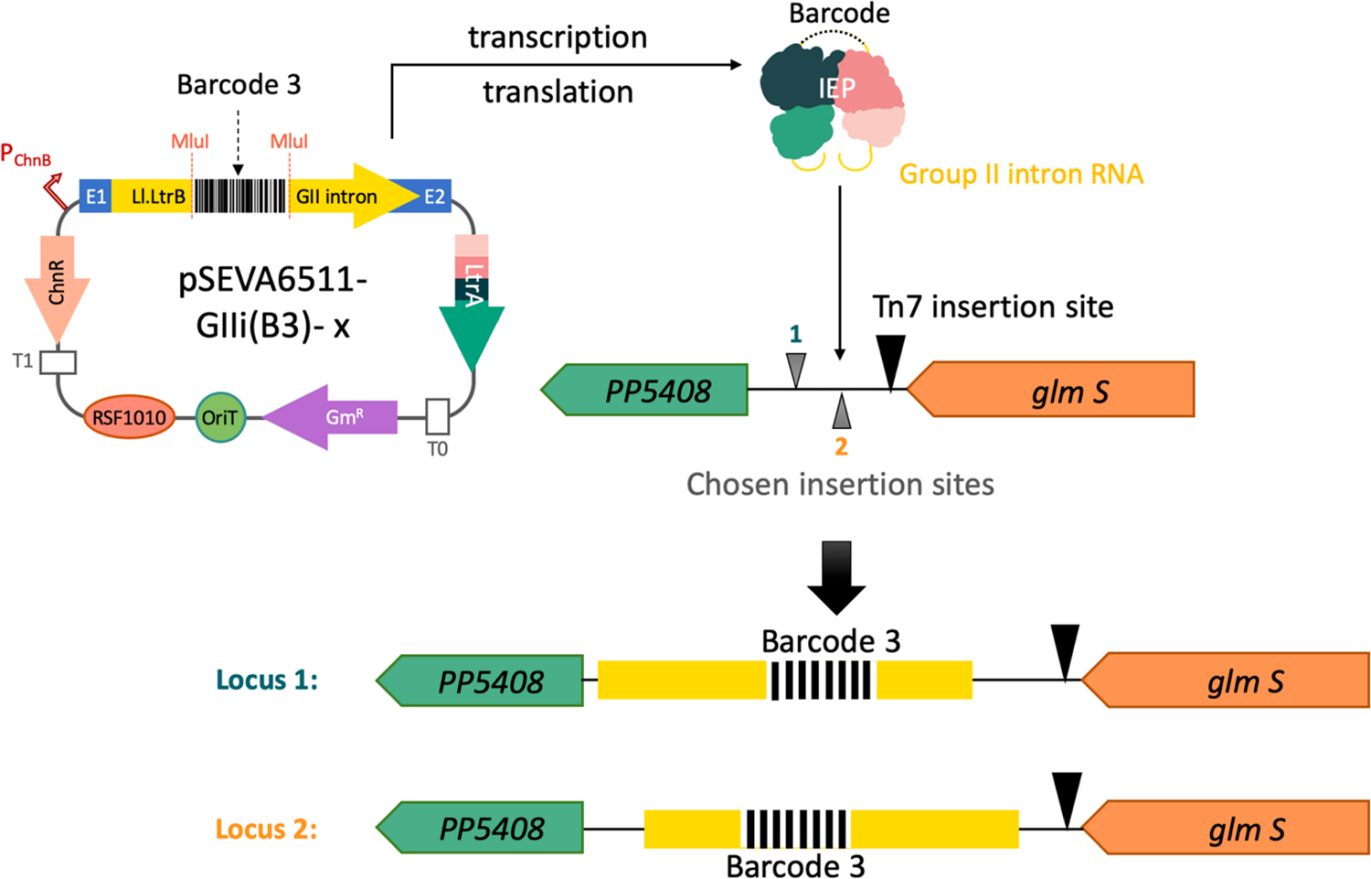
Application of the Ll.LtrB group II intron for delivery of specific genetic barcodes to the genome of *P. putida* KT2440. Organization of pSEVA6511-GIIi (B3) variants is shown with the intron retargeted toward Locus 1 (*x* = 37s) or Locus 2 (*x* = 95a). Selection of the insertion loci for Ll.LtrB::B3 is in the vicinity of the Tn7-insertion site (black triangle). Two different insertion points (gray triangles) were chosen for the insertion list generated on the Clostron website,^24^ and Ll.LtrB::B3 was retargeted to both sites accordingly. The recognition site in Locus 1 (green) is located in the sense strand, while Locus 2 (orange) is present in the antisense strand of the *P. putida* genome. Ll.LtrB::B3 insertion would generate two different genotypes depending on the locus being targeted in each case.

To exploit the Ll.LtrB/LtrA-based vector platform described above for targeting short sequences to the *P. putida* genome, a specific barcode generated with the CellRepo algorithm^61^ was created with synthetic oligonucleotides (Supplementary Figure S4) and then cloned as a cargo of LI.LtrB in pSEVA6511-GIIi to generate pSEVA6511-GIIi (B3). Next, we searched for adequate targeting loci in the genome of *P. putida*. As barcodes are meant to link a strain to its digital data, they need to be included in a stable and permissive genetic locus,^61,62^ such as intergenic regions close to essential genes. The context close to *glmS* (close to the *att* Tn7 site, Figure 6) was thus selected as a good candidate for the insertion of Ll.LtrB::B3. The Clostron algorithm^24^ (http://www.clostron.com/) was used to survey the intergenic region between *PP5408* and *glmS* to identify optimal targets. Two loci were picked from the retrieved list that were compatible with CRISPR/Cas9 counterselection (Figure 6 and Supplementary Figure S5), i.e., they contained a nearby PAM sequence in their vicinity in the correct orientation.

**Figure 7:**
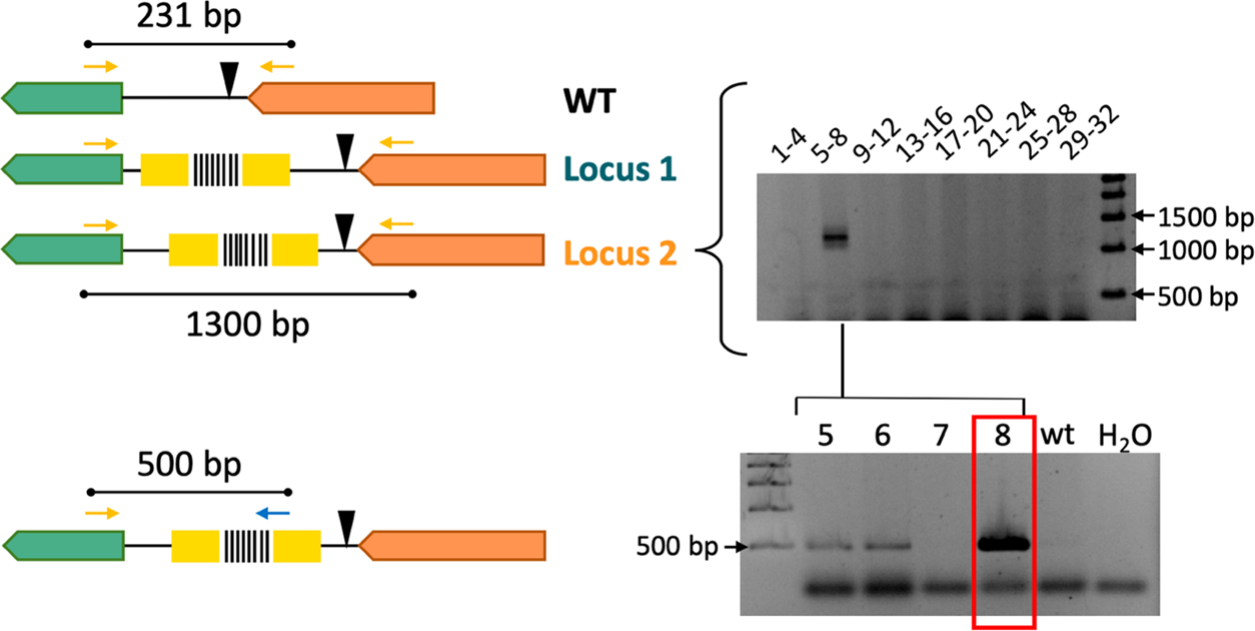
Delivery of Ll.LtrB::B3 containing a barcode in the *P. putida* KT2440 genome. A first pool PCR was set to detect successful Ll.LtrB::B3 insertions in either Locus 1 (green) or Locus 2 (orange). The top gel shows the amplification found with a pool PCR using primers flanking the insertion at Locus 2. The bottom gel shows the second PCR of individual colonies from the corresponding pool to find the barcoded clone. In this case, a primer annealing inside the barcode (pbarcode universal) and another annealing inside the *PP5408* gene were used. WT: wild-type, Locus 1: 36,37s insertion site, Locus 2: 94,95a insertion site. *PP5408* (green gene), *glmS* (orange gene).

Then, the retargeting region of Ll.LtrB was separately engineered to these two loci, giving rise to pSEVA6511-GIIi(B3)-37s and pSEVA6511-GIIi(B3)-94a, respectively (Figure 6). In parallel, specific CRISPR spacers for either locus were designed and tested to measure the efficiency of CRISPR/Cas9 cleavage, which was >90% in both cases (Supplementary Figure S6). After these two components were ready, the same insertion protocol we used before for *luxC* fragments was adopted for the delivery of the barcodes. Thereby, the obtained colonies were directly checked through pool PCR reactions for correct barcode insertion, as no phenotype change was expected after the insertion of the cargo-containing Ll.LtrB. Additional PCRs were performed to secure the purity of isolated colonies (data not shown), and barcode integrity was confirmed through sequencing. Only in the case of Locus 2, *bona fide* insertions were found to bear the corresponding barcode sequence (Figure 7). Details about the final barcoded strain can be found in the public CellRepo repository: https://cellrepo.ico2s.org/repositories/93?branch_id=139&locale=en.

## Conclusions

While *P. putida* KT2440 has made evident its utility in biotechnological applications, there is still a need to develop new tools that can be used to modify this bacterium and broaden its applicability. Moreover, testing these broad-host-range tools in *P. putida* showcases their potential for modifying a suite of other Gram-negative bacteria of interest, especially those deficient in homologous recombination. This work is an attempt to expand the number of genetic assets that can be exploited to insert sequences of certain lengths at specific genomic regions, in this case using group II introns. Given the functionality of these retroelements through virtually all of the evolutionary trees^22,63^ and the orthogonality of their DNA insertion mechanism,^24,30,38^ we argue that they are ideal devices for the incorporation of standardized short sequences in a wide variety of biological destinations. One specific application is the insertion of unique identifiers for barcoding strains^39^ and other live items of biotechnological interest, for the sake of traceability and securing intellectual property.^64^ While the SEVA-based system described here is ready to use in *Pseudomonas* and other Gram-negative bacteria, the minimal delivery device formed by the Ll.LtrB element and the LtrA protein complex can be easily moved to any other platform optimized for other recipients. We argue that this work contributes to the developing collection of trans-kingdom genetic tools for the insertion of DNA sequences in a range of biological systems while using the same delivery mechanism.

## Methods

### Bacterial Strains and Media

*E. coli* CC118 strain [Δ(*ara-leu*), *araD139*, Δ*lacX74*, *galE*, *galK phoA20*, *thi-1*, *rpsE*, *rpoB*, *argE* (Am), *recA1*, OmpC^+^, OmpF^+^] was used for plasmid cloning and propagation and BL21DE3 strain [*fhuA2*, [lon], *ompT*, *gal*, (λ DE3), [dcm], Δ*hsdS*; (λ DE3) = λ s*Bam*HIo Δ*Eco*RI-B int::(*lacI*::*PlacUV5*::T7 gene1) i21 Δnin5] for intron mobility assays in *E. coli*. *P. putida* KT2440 and its derivative Δ*recA* were used to assess intron mobility in this species. Luria-Bertani (LB) medium was used for general growth and was supplemented when needed with kanamycin (Km; 50 μg/mL), ampicillin (Ap; 150 μg/mL for *E. coli* and 500 μg/mL for *P. putida*), gentamycin (Gm; 10 μg/mL for *E. coli* and 15 μg/mL for *P. putida*), and/or streptomycin (Sm; 50 μg/mL for *E. coli* and 100 μg/mL for *P. putida*). For solid plates, LB medium was supplemented with 1.5% agar (w/v). In specific cases for *P. putida*, M9 minimal medium [6 g/L Na_2_HPO_4_, 3 g/L KH_2_PO_4_, 1.4 g/L (NH_4_)_2_SO_4_, 0.5 g/L NaCl, 0.2 g/L MgSO_4_·7H_2_O] supplemented with sodium citrate at 0.2% (w/v) as the carbon source was used instead. X-gal (5-bromo-4-chloro-3-indolyl-β-d-galactopyranoside) was added at a final concentration of 30 μg/mL to carry out blue/white colony screening. Moreover, different inducers were added to media when necessary: isopropyl-1-thio-*b*-galactopyranoside (IPTG) and cyclohexanone at 1 mM unless stated otherwise.

### Plasmid Construction

The complete sequence encoding the T7 promoter, Ll.LtrB intron, and LtrA protein was amplified from the commercial plasmid pACD4K-C (TargeTron gene knockout system, Sigma-Aldrich) with primers pGIIintron_fwd and rev (Supplementary Table S1). The amplified fragment was then digested with *Pac*I and *Spe*I restriction enzymes and cloned into a similarly digested pSEVA427, yielding pSEVA421-GIIi (Km). The *lacUV5* promoter along with T7 RNA polymerase (T7 RNAP) sequences was amplified from pAR1219 (Merck) with primers pAR1219_fwd and rev, *Pac*I/*Spe*I digested, and cloned into corresponding sites of pSEVA131, generating pSEVA131-T7RNAP, necessary for the transcription of the Ll.LtrB intron from the T7 promoter. To eliminate the retrotransposition-activated selectable marker (RAM) present inside Ll.LtrB, pSEVA421-GIIi (Km) was digested with the *Mlu*I restriction enzyme and then directly ligated and transformed to obtain pSEVA421-GIIi. To change the expression system and simplify the intron expression mechanism, only Ll.LtrB (with or without RAM) and LtrA sequences were extracted by *Hin*dIII/*Spe*I digestion of pSEVA421-GIIi and cloned into pSEVA2311, giving rise to pSEVA2311-GIIi (Km) and pSEVA2311-GIIi, respectively. These plasmids have both the Ll.LtrB intron and LtrA expression controlled under the ChnR-P_ChnB_ promoter. Finally, to assemble an expression plasmid compatible with the CRISPR/Cas9 system described previously,^36^ it was necessary to modify both the origin of replication and the antibiotic resistance gene. For that, the ChnR-P_ChnB_ promoter, Ll.LtrB (with or without RAM), and LtrA sequences were extracted by digestion with *Pac*I/*Spe*I enzymes and cloned into pSEVA651 equivalent sites to obtain pSEVA6511-GIIi (Km) and pSEVA6511-GIIi. The CRISPR/Cas9 counterselection approach used in this work was described in ref (36) and was based on plasmids pSEVA421-Cas9tr and pSEVA231-CRISPR. pSEVA231-C-pyrF1 was generated and described in the same work. The rest of the spacers for counterselection were designed manually and cloned into *Bsa*I-digested pSEVA231-CRISPR, following the protocol explained in the same paper. The resulting plasmids were named pSEVA231-C-37s and pSEVA231-C-94a.

### Retargeting of the Ll.LtrB Intron

Retargeting of the Ll.LtrB intron was performed by adapting the Targetron protocol from Sigma-Aldrich. First, the Clostron platform^24^ (http://www.clostron.com/) was used to design primers pIBS-X, pEBS1d-X, pEBS2-X, and pEBSuniversal (depending on the insertion target; Supplementary Table S1) with corresponding target sequences as query (*lacZ* gene in *E. coli*; *pyrF* gene and *PP5408*-*glmS* region in *P. putida*). From the output list, the best-ranked targets compatible with CRISPR/Cas9 technology were selected in each case. This means targets with PAM sequences (5′-NGG-3′ in the case of the *Streptococcus pyogenes* system) closest to the insertion site of the intron were chosen. Afterward, Clostron-designed oligonucleotides for each target were used in a SOEing PCR^65^ with pACD4K-C as a template to yield a 350 bp fragment. For the cloning of this amplicon, different strategies were adopted according to the final recipient plasmid. For the retargeting of pSEVA421-GIIi and its derivatives, the fragment was digested with *Bsr*GI/*Hin*dIII restriction enzymes and ligated into the linearized recipient plasmid. For retargeting of pSEVA2311-GIIi and pSEVA6511 derivatives, Gibson assembly^66,67^ was chosen as the cloning procedure since an additional *Bsr*GI restriction site was present in the pChnB promoter. Primers pRetarget-fwd and rev were used to reamplify the SOEing amplicon and add the corresponding homologous sequences to directly assemble the fragment to *Hin*dIII/*Hpa*I-digested pSEVA2311/6511-GIIi.

### Insertion of Exogenous Sequences Inside Ll.LtrB

All exogenous sequences inserted inside the Ll.LtrB intron were cloned into the *Mlu*I site present in the intron sequence. In the case of the insert to be delivered, two strategies were followed: the *luxC* gene from the lux operon was employed as a template for the generation of fragments of different sizes (from 150 to 1050 bp with a difference of 150 bp each). Primer pLux_fwd in combination with primer pLux1–7_rev (Supplementary Table S1) was used in a PCR step to generate each fragment using as template pSEVA256. Each amplicon was then digested with *Mlu*I and cloned into linearized pSEVA6511-GIIi-pyrF. The sense orientation of each fragment was confirmed by sequencing. Barcode sequences were created on the CellRepo website (https://cellrepo.herokuapp.com) with an algorithm that provides universally unique identifiers (UUIDs).^61^ This provides the possibility to produce a large library of barcodes that are randomly generated and unique. After selecting one specific barcode, a BLAST search was done to make sure there was no other region with high similarity in the genome of *P. putida*. Once a barcode was verified, it was generated by a PCR step with 119-mer oligonucleotides bearing 30 overlapping nucleotides at 3′. These primers also included 30 nucleotides complementary to the recipient vector at 5′, so a Gibson assembly reaction^66,67^ could be performed after amplification with *Mlu*I-digested pSEVA6511-GIIi.

### Interference Assay of Spacers 37s and 94a

*P. putida* KT2440 strain harboring pSEVA421-Cas9tr was grown overnight, and electrocompetent cells were prepared by washing cells with 300 mM sucrose a total of five times. The final pellet was resuspended in 400 μL and then split into 100 μL aliquots. One hundred nanograms of pSEVA231-CRISPR (control), pSEVA231-C-37s, or pSEVA231-C-94a (Supplementary Table S2) was electroporated into respective aliquots. Transformed bacteria were grown in LB/Sm for 2 h at 30 °C and serial dilutions were then plated on LB/Sm to test viability and on LB/Sm/Km plates to assess the efficiency of cleavage. After counting CFUs under both conditions, the ratio of transformation efficiency was calculated by dividing the CFUs on LB/Sm/Km plates by CFUs on LB/Sm plates and normalized to 10^9^ cells.

### Ll.LtrB Insertion Assay in *E. coli*

Briefly, cells harboring the corresponding pSEVA-GIIi derivative plasmid were grown in LB supplemented with the corresponding antibiotics. When an OD of 0.2 was reached, the right inducer was added to the medium and the cells were incubated at 30 °C for different periods (from 30 min to 4 h depending on the expression system). When IPTG was used, the cells were washed and recuperated in fresh media after the induction period of 1 h at 30 °C. Finally, serial dilutions were plated to assess viability and intron insertion efficiency on selective media as indicated in each case.

### Ll.LtrB Insertion Assay in *P. putida*

The same protocol described above for *E. coli* was used with *P. putida* strains, with the only difference that the induction time was 2 or 4 h (depending on the expression system), and no recovery was performed after 4 h induction. Also, as *pyrF* gene was the target of Ll.LtrB insertion, the cells were plated on M9 minimal media supplemented with only 20 μg/mL uracil (Ura) to assess viability on uracil and 250 μg/mL 5-fluoroorotic acid (5FOA) to counterselect *pyrF*-disruption mutants and make easier the identification of insertion events. An average of 50 colonies per condition were patched on minimal media with and without uracil to assess the proportion of uracil auxotrophs among the 5FOA^R^ colonies.

### CRISPR/Cas9 Counterselection Assay in *P. putida* KT2440 and KT2440 Δ*recA*

When CRISPR/Cas9 counterselection was to be applied, the protocol was modified to simplify the process. The cells harboring both pSEVA421-Cas9tr and pSEVA6511-GIIi derivatives were grown overnight at 30 °C. The next day, 1 mM cyclohexanone was added to the culture, and the cells were induced for 4 h at 30 °C. After this incubation, 1 mL of cells was plated on M9 minimal media supplemented with Ura and 5FOA to assess the native efficiency of insertion in this condition. Later, the cells were made electrocompetent, and 100 ng of pSEVA231-CRISPR or pSEVA231-C-spacer (pyrF1, 37s or 94a depending on the experiment) was electroporated. Finally, the cells were recovered in LB/Sm for 2 h at 30 °C, after which serial dilutions were plated on LB/Sm (to assess viability) and LB/Sm/Km (to assess counterselection efficiency).

### Calculation of Merged Targetrons/CRISPR/Cas9 Efficiency

For the generation of this ratio, the normalized frequency of Sm^R^ Km^R^ CFU obtained per 10^9^ viable cells (Sm^R^) after transforming pSEVA231-CRISPR or pSEVA231-C-pyrF1 was first calculated with the formula
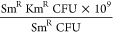


Next, the ratio of counterselection efficiency (*R*) was calculated by dividing the normalized Sm^R^ Km^R^ CFU obtained with pSEVA231-C-pyrF1 by the one calculated for pSEVA231-CRISPR.
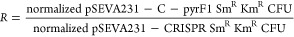


Finally, the efficiency of the merged Ll.LtrB insertion and CRISPR/Cas9 counterselection was obtained after multiplying the percentage of positive clones verified through PCR in the pSEVA231-C-pyrF1 condition. The final ratio was plotted as a percentage
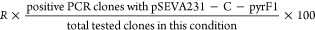


These formulae were used with each cargo size and in each replicate individually. The error and standard deviation were calculated based on the efficiency of targetron + CRISPR system ratio of each replicate using GraphPad Prism 6 (https://www.graphpad.com).

### Analysis of Ll.LtrB Insertion by Colony PCR

Ll.LtrB integrations were studied by colony PCR to check the presence or absence of the intron in the correct loci. Two possible reactions were used: in one, primers flanking the insertion site to amplify the whole group II intron were used. The product of this PCR would be composed of the intron sequence and the amplified flanking regions. In the second, one primer was annealed in the target locus and the other inside the intron sequence; consequently, a PCR product was only obtained when the Ll.LtrB intron was present. In the case of barcode delivery, pool PCR reactions with a total of four colonies per reaction were first performed, followed by PCR of individual colonies in positive pools. PCRs were analyzed by electrophoresis on agarose gel and 1× TAE (Tris-Acetate-EDTA). EZ Load 500 bp Molecular Ruler (Brio-Rad) was the DNA ladder in all gels.

## Supporting Information Available

List of oligonucleotides used in this study (Supplementary Table S1); list of plasmids used in this work (Supplementary Table S2); insertion frequencies of Ll.LtrB::Lux1 and Ll.LtrB::Lux4 in *P. putida* KT2440 WT and Δ*recA* with no CRISPR/Cas9-mediated counterselection (Supplementary Table S3); insertion frequency of Ll.LtrB::LuxN intron in *P. putida* KT2440 WT with 5FOA CRISPR/Cas9-mediated counterselection (Supplementary Table S4); insertion frequency of Ll.LtrB::LuxN intron in *P. putida* KT2440 Δ*recA* with 5FOA CRISPR/Cas9-mediated counterselection (Supplementary Table S5); construction and verification of intron delivery plasmids compatible with CRISPR/Cas9-mediated counterselection (Supplementary Methods); pSEVA plasmids for the expression of the Ll.LtrB intron in a wide range of Gram-negative bacteria (Supplementary Figure S1); preliminary approach to assess size-restriction of intron-mediated delivery using *luxC* fragments as a cargo and 5FOA counterselection in log-phase induced cells (Supplementary Figure S2); total intron insertion frequency without differentiating the cargo that was being delivered (Supplementary Figure S3); barcode generation with a PCR using 3′-overlapping 119-mer oligonucleotides (Supplementary Figure S4); application of targetrons as a barcode delivery system (Supplementary Figure S5); and design and test of Locus 1 and 2 spacers for CRISPR/Cas9-mediated counterselection of Ll.LtrB::B3 group II intron (Supplementary Figure S6) (PDF)
