# Supplementary material for: Targetron-Assisted Delivery of Exogenous DNA Sequences into Pseudomonas putida through CRISPR-Aided Counterselection
Source: ACS Synth Biol. 2021 Oct 2;10(10):2552–65. doi: 10.1021/acssynbio.1c00199 (PMC8524655; doi:10.1021/acssynbio.1c00199)
Supplement: Supplementary file 1 — sb1c00199_si_001.pdf [file sb1c00199_si_001.pdf]

SUPPORTING INFORMATION

Targetron-assisted delivery of exogenous DNA sequences into *Pseudomonas putida* through CRISPR-aided counterselection

by

Elena Velázquez[1], Yamal Al-Ramahi[1], Jonathan Tellechea-Luzardo[2], Natalio Krasnogor[2] and Víctor de Lorenzo[1]*

[1] Systems and Synthetic Biology Department, Centro Nacional de Biotecnología (CNB-CSIC), Campus de Cantoblanco, Madrid 28049, Spain. [2]Interdisciplinary Computing and Complex Biosystems (ICOS) Research Group, Newcastle University, Newcastle Upon Tyne NE4 5TG, U.K.

SUPPLEMENTARY TABLES

Supplementary Table S1. List of oligonucleotides used in this study

Name	Sequence (5' → 3')*	Tm (°C)	Use
**pS1**	AGG GCG GCG GAT TTG TCC	71.7	Sequencing of pSEVA from Terminator T1
**pS2**	GCG GCA ACC GAG CGT TC	70.6	Sequencing of pSEVA from Terminator T0
**T7p**	TAA TAC GAC TCA CTA TAG GG	50.9	Sequencing from T7 promoter region
**pLacZ_F**	CTC GTT GCT GCA TAA ACC GAC	66.6	PCR to verify Ll.LtrB insertion in locus 1063a of *lacZ* gene
**pLacZ_R**	GAT GGA CCA TTT CGG CAC AGC	70.9	
**pGIIintron_fwd**	TTA TTA TAT TAA TTA ACG CGA AAT TAA TAC GAC TCA C	66.1	Construction of pSEVA421-GIIi
**pGIIintron_rev**	TTA TTA TAA CTA GTG GTG CGG ACT GTT GTA ACT C	68.4	
**pSEVA427-out-SpeI**	TGC GTT CGG TCA AGG TTC	65.1	Sequencing pSEVA421-GIIi
**pAR1219_fwd**	TTA TTA TAT TAA TTA ACA GAT CCC GGA CAC CAT CGA ATG GCG CAA AAC C	81.4	Construction of pSEVA131-T7RNAP
**pAR1219_rev**	TTA TTA TAA CTA GTG GCG TTA CGC GAA CGC GAA GTC CGA CTC TAA GAT G	81.4	
**EBS universal**	CGA AAT TAG AAA CTT GCG TTC AGT AAA C	66.2	Universal primer for Ll.LtrB intron retargeting
**pyrF_165a-EBS1d**	CAG ATT GTA CAA ATG TGG TGA TAA CAG ATA AGT CGA AGC CCT TAA CTT ACC TTT CTT TGT	80.3	Ll.LtrB retargeting for insertion in locus 165a (antisense orientation) of *Pseudomonas putida pyrF* gene. In combination with primer EBS universal
**pyrF_165a-IBS**	AAA AAA GCT TAT AAT TAT CCT TAC ACT TCG AAG CCG TGC GCC CAG ATA GGG TG	82.9	
**pyrF_165a-EBS2**	TGA ACG CAA GTT TCT AAT TTC GGT TAA GTG TCG ATA GAG GAA AGT GTC T	79.6	

**pyrF_F**	ACT TGC CAA GAG ACC CTG	60.5	Confirmation of insertion of Ll.LtrB intron in locus 165a
**pyrF_R**	TCA CGC CAA TCA GCA ACG	67.4	
**pRetarget-fwd**	AGA GTC GAC CTG CAG GCA TGC AAG CTT ATA ATT ATC CTT A	77.6	Construction of retargeted pSEVA2311-GIIi and pSEVA6511-GIIi derivatives
**pRetarget-rev**	GTT CTC CTA CAG ATT GTA CAA ATG TGG TGA TAA CAG ATA	73.0	
**Barcode_3_F**	CGT CCA GAT ATT TAT TAC GTG GCG ACG CGT TGG ACA TAC ATA GTA TAC TCT GGT GTT GAA GTT TCA AGG TTT TAT CCG TAG GTT CAA CTG CGT TGG GAG TGG GCA AGG GGG AAT GGG CA	94.4	Construction of pSEVA6511-GIIi(B3)
**Barcode_3_R**	TTT CGC TAT CAT TGC CAT TTC CCA ACG CGT CCA AAG GCA AAC TTG TCA AGT CTA TGG ATA ACC TAC TAA CCC CAT TGT TTT TAT ATC TAT GCC CAT TCC CCC TTG CCC ACT CCC AAC GC	94.2	
**spacer 94a-F**	[Phos][a)] AAA CTG TCG CAG GAG CCG GTG AGG TCT GTC TCA TG	83.5	Construction of pSEVA231-C-94a
**spacer 94a-R**	[Phos][a)] AAA ACA TGA GAC AGA CCT CAC CGG CTC CTG CGA CA	83.6	
**spacer 37s-F**	[Phos][a)] AAA CCA TGG ATT GCT GGT CCC TAG GGA GCA GGA AG	81.4	Construction of pSEVA231-C-37s
**Spacer 37s-R**	[Phos][a)] AAA ACT TCC TGC TCC CTA GGG ACC AGC AAT CCA TG	80.1	
**37s-IBS**	AAA AAA GCT TAT AAT TAT CCT TAT AGG GCG CAG GAG TGC GCC CAG ATA GGG TG	83.1	Ll.LtrB retargeting for insertion in locus 37s (sense orientation) between *PP5408* and *glmS* genes in *P. putida*. In combination with primer EBS universal
**37s-EBS1d**	CAG ATT GTA CAA ATG TGG TGA TAA CAG ATA AGT CGC AGG AAA TAA CTT ACC TTT CTT TGT	79.9	
**37s-EBS2**	TGA ACG CAA GTT TCT AAT TTC GGT TCC CTA TCG ATA GAG GAA AGT GTC T	80.7	
**94a-IBS**	AAA AAA GCT TAT AAT TAT CCT TAG GTG ACG TCT GTG TGC GCC CAG ATA GGG TG	82.3	Ll.LtrB retargeting for insertion in locus 94a (antisense orientation)

**94a-EBS1d**	CAG ATT GTA CAA ATG TGG TGA TAA CAG ATA AGT CGT CTG TCT TAA CTT ACC TTT CTT TGT	78.8	between PP5408 and *glmS* genes in *P. putida*. In combination with primer EBS universal
**94a-EBS2**	TGA ACG CAA GTT TCT AAT TTC GAT TTC ACC TCG ATA GAG GAA AGT GTC T	80.6	
**pbarcodes universal**	TGG ACA TAC ATA GTA TAC TCT GGT G	58.6	Sequencing of Barcodes and PCR for confirmation of intron insertion
**pbarcodes GIIi reverse**	ACA CAA TAA CTG TAC CCC TTT GCC	65.7	
**649-Tn7-F**	CGA TTC ATC AGG TTG GAT TCG	66.4	PCR confirmation of Ll.LtrB insertion in *PP5408-glms* intronic region
**418-Tn7-R**	AAT CTG GCC AAG TCG GTG AC	66.3	
**pLux_fwd**	TTA TTA TAC GCG TAT GAC TAA AAA AAT TTC ATT CAT TAT TAA CGG	71.7	Amplification of *luxC* gene to produce cargos of different sizes
**pLux1_rev**	TTA TTA TAC GCG TAT TAC AAT CAA TAA TGT TTT TTA CAT GAG AGT C	71.1	
**pLux2_rev**	TTA TTA TAC GCG TCT CTA GCT TAG CCA TTT CTT CTG	71.5	
**pLux3_rev**	TTA TTA TAC GCG TCA GAT GTA CAG ATT TAC CTT TC	68.8	
**pLux4_rev**	TTA TTA TAC GCG TCG GAT GAT TAG GGT CTA C	69.8	
**pLux5_rev**	TTA TTA TAC GCG TAT CAG CAT AAG ATG GCG	70.5	
**pLux6_rev**	TTA TTA TAC GCG TAT GAT TTC CCA TGT AAT ATA TGT TTT G	70.4	
**pLux7_rev**	TTA TTA TAC GCG TAT GAT TTC CCA TGT AAT ATA TGT TTT G	68.2	
**pgII_cargo_fwd**	TAG TAG TCT GAG AAG GGT AAC G	52.8	Sequencing Lux inserts in Ll.LtrB intron. Confirmation of Ll.LtrB insertion in combination with primers pyrF_F and pyrF_R, respectively.
**pGIIi_cargo_rev**	GTA TAC GGC TCT GTT ATT GTT C	51.0	

a) [Phos]: Phosphorothioate group

Supplementary Table S2. List of plasmids used in this work

Plasmid	Description	Reference
**pAR1219**	Expression plasmid carrying T7 RNA polymerase gene (bacteriophage gene 1) under control of *lac*UV5 promoter; oriV(pMB1); Ap^R	Merck (Sigma-Aldrich)
**pACD4K-C**	Expression plasmid carrying Ll.LtrB intron (bearing Km^R *Retrotransposition-Activated selectable Marker*, RAM) under control of T7 promoter and retargeted to insert into locus 1063 of *E. coli lacZ* gene in the antisense orientation; oriV(p15A); Cm^R	Merck (Sigma-Aldrich)
**pSEVA231-CRISPR**	pSEVA231 derivative bearing CRISPR array; oriV (pBBR1); Km^R	[1]
**pSEVA421-Cas9tr**	pSEVA421 derivative bearing the cas9 gene and tracrRNA; oriV (RK2); Sm^R/Sp^R	[1]
**pSEVA131**	Standard SEVA expression vector; oriV(pBBR1); Ap^R	[2]
**pSEVA427**	Standard SEVA expression vector; oriV(pBBR1); *gfp* cargo; Sm^R/Sp^R	[2]
**pSEVA131-T7RNAP**	pSEVA131 derivative with T7 RNA polymerase gene expression under *lac*UV5 promoter control	This work
**pSEVA421-GIIi(Km)**	pSEVA427 derivative expressing Ll.LtrB group II intron under T7 promoter control. Ll.LtrB bearing the Km^R RAM and retargeted to insert into 1063 locus of *E. coli lacZ* gene in antisense orientation.	This work
**pSEVA421-GIIi-pyrF**	pSEVA421-GIIi(Km) derivative with empty Ll.LtrB intron and retargeted to insert into 165 locus of *P. putida pyrF* gene in antisense orientation.	This work
**pSEVA421-GIIi(Km)-pyrF**	pSEVA421-GIIi(Km) derivative with Ll.LtrB intron bearing Km^R RAM and retargeted to insert into 165 locus of *P. putida pyrF* gene in antisense orientation.	This work
**pSEVA2311**	Standard SEVA expression vector; oriV(pBBR1); ChnR-PChnB, cyclohexanone-responsive expression plasmid; Km^R	[3,4]
**pSEVA2311-GIIi(Km)**	pSEVA2311 derivative with Ll.LtrB intron under ChnR-PChnB promoter. Ll.LtrB intron bearing Km^R RAM and retargeted to insert into 1063 locus of *E. coli lacZ* gene in antisense orientation.	This work
**pSEVA2311-GIIi-pyrF**	pSEVA2311-GIIi(Km) derivative with empty (no RAM) Ll.LtrB intron retargeted to insert into 165 locus of *P. putida pyrF* gene in antisense orientation.	This work

**pSEVA651**	Standard SEVA expression vector; oriV(RSF1010); Gm^R	2
**pSEVA6511-GIIi(Km)**	pSEVA651 derivative with ChnR-PChnB promoter and Ll.LtrB intron from pSEVA2311-GIIi(Km) as a PacI/SpeI insert.	This work
**pSEVA6511-GIIi**	pSEVA6511-GIIi(Km) derivative with no Km^R RAM (empty Ll.LtrB)	This work
**pSEVA6511-GIIi-pyrF**	pSEVA6511-GIIi derivative retargeted to insert into 165 locus of *P putida pyrF* gene.	This work
**pSEVA231-C-pyrF1**	pSEVA231-CRISPR derivative with *pyr*F spacer cloned into BsaI restriction sites.	1
**pSEVA256**	Standard SEVA expression vector; oriV(RSF1010); *luxCDABE* as cargo; Km^R	2
**pSEVA6511-GIIi(Lux1)-pyrF**	pSEVA6511-GIIi-pyrF derivative with Lux1 (150 bp) insert in sense orientation	This work
**pSEVA6511-GIIi(Lux2)-pyrF**	pSEVA6511-GIIi-pyrF derivative with Lux2 (300 bp) insert in sense orientation	This work
**pSEVA6511-GIIi(Lux3)-pyrF**	pSEVA6511-GIIi-pyrF derivative with Lux3 (450 bp) insert in sense orientation	This work
**pSEVA6511-GIIi(Lux4)-pyrF**	pSEVA6511-GIIi-pyrF derivative with Lux4 (600 bp) insert in sense orientation	This work
**pSEVA6511-GIIi(Lux5)-pyrF**	pSEVA6511-GIIi-pyrF derivative with Lux5 (750 bp) insert in sense orientation	This work
**pSEVA6511-GIIi(Lux6)-pyrF**	pSEVA6511-GIIi-pyrF derivative with Lux6 (900 bp) insert in sense orientation	This work
**pSEVA6511-GIIi(Lux7)-pyrF**	pSEVA6511-GIIi-pyrF derivative with Lux7 (1050 bp) insert in sense orientation	This work
**pSEVA231-C-37s**	pSEVA231-CRISPR derivative with 37s spacer cloned into BsaI restriction sites	This work
**pSEVA231-C-94a**	pSEVA231-CRISPR derivative with 94a spacer cloned into BsaI restriction sites	This work
**pSEVA6511-GIIi(B3)-37s**	pSEVA6511-GIIi derivative retargeted to insert into locus 37 (in sense orientation) between *PP5408* and *glmS* genes in *P. putida* and bearing barcode 3 as a MluI insert.	This work
**pSEVA6511-GIIi(B3)-94a**	pSEVA6511-GIIi derivative retargeted to insert into locus 94 (in antisense orientation) between *PP5408* and *glmS* genes in *P. putida* and bearing barcode 3 as a MluI insert.	This work

Supplementary Table S3. Preliminary appraisal of the size limit of intron-mediated delivery of DNA fragments. Insertion frequencies of Ll.LtrB::Lux1 and Ll.LtrB::Lux4 in *P. putida* KT2440 WT and Δ*recA* with no CRISPR/Cas9-mediated counterselection.

	From Ura (+ intron/total re-streaks)	From FOA (+ intron/total re-streaks)	
**Lux1**	1/105 (0.95%)	17/108 (16%)	wt
**Lux4**	0/104	2/50 (4%)	
**Lux1**	0/101	10/106 (9.4%)	ΔrecA
**Lux4**	0/102	0/61	

Supplementary Table S4. Insertion frequency of Ll.LtrB::LuxN intron in *P. putida* KT2440 WT with 5FOA and CRISPR/Cas9-mediated counterselection.

		Replicate 1	Replicate 2	Replicate 3	Total
**5FOA**	LuxØ	0/58	1/53	0/51	1/162
	Lux1	0/60	0/56	0/56	0/172
	Lux2	0/60	0/31	0/51	0/142
	Lux3	0/59	0/56	0/55	0/170
	Lux4	0/60	0/49	0/54	0/163
**231-CRISPR**	LuxØ	0/10	2/48	4/46	6/104
	Lux1	0/56	0/2	0/104	0/162
	Lux2	0/42	0/53	0/107	0/202
	Lux3	0/56	0/56	0/106	0/218
	Lux4	0/48	0/52	0/104	0/204
**231-C-pyrF1**	LuxØ	13/18	7/56	17/40	37/114
	Lux1	1/53	0/24	0/10	1/87
	Lux2	0/56	0/51	-	0/107
	Lux3	0/57	0/50	0/96	0/203
	Lux4	0/56	0/48	0/87	0/191

*Number of positive intron-insertion colonies confirmed through PCR divided by total of screened colonies in each replicate.

Supplementary Table S5. Insertion frequency of Ll.LtrB::LuxN intron in *P. putida* KT2440 Δ*recA* with 5FOA and CRISPR/Cas9-mediated counterselection.

		Replicate 1	Replicate 2	Replicate 3	Total
**5FOA**	LuxØ	0/35	3/51	5/28	8/114
	Lux1	0/50	0/56	0/56	0/162
	Lux2	0/38	0/50	0/49	0/137
	Lux3	0/40	0/54	-	0/94
	Lux4	0/56	0/51	-	0/107
**231-CRISPR**	LuxØ	1/51	3/30	1/49	5/130
	Lux1	1/55	1/34	0/50	2/139
	Lux2	0/56	0/35	0/53	0/144
	Lux3	0/54	0/56	-	0/110
	Lux4	0/53	0/39	-	0/92
**231-C-pyrF1**	LuxØ	1/40	7/40	4/40	11/120
	Lux1	2/48	1/40	10/40	13/128
	Lux2	7/15	1/40	2/3	10/58
	Lux3	3/23	3/10	-	6/33
	Lux4	2/8	1/1	-	3/9

*Number of positive intron-insertion colonies confirmed through PCR divided by total of screened colonies in each replicate.

SUPPLEMENTARY METHODS

Construction and verification of intron-delivery plasmids compatible with CRISPR/Cas9-mediated counterselection. pSEVA6511-GIIi and pSEVA6511-GIIi (Km) (Supplementary Fig. S1G and H) are broad-host-range plasmids, have a medium copy number in *E. coli* and a relatively high copy number in *P. putida* (RSF1010 *oriV* from SEVA plasmids [2,5–7]), a gentamycin resistance gene and a *lacZ*-retargeted Ll.LtrB and LtrA controlled by the ChnR/P_{ChnB} promoter. The only difference between them was the absence or presence of the RAM. The efficacy of the approach was first tested in *E. coli* BL21DE3 (Figure below A-C), where we were able to identify insertion mutants generated by the introns borne by either plasmid. In the case of pSEVA6511-GIIi, frequencies were enough to spot white colonies on plates supplemented with X-gal even without any selection. This was in contrast with the case of pSEVA6511-GIIi (Km), which produced white colonies only when Km was added to plates. This supports the notion that the presence of the RAM inside Ll.LtrB helps selection of insertions while it interferes with the efficacy of the process (insertion frequencies decreased from ~10^{-2} to ~10^{-7}; Figure below A, B). Note also the notable decline of insertion efficiencies compared to those brought about by plasmid pSEVA421-GIIi (Km) as shown in B. This can be blamed on the adoption of a σ^{70}-dependent promoter. P_{ChnB} is transcribed by the housekeeping RNAP in plasmids pSEVA6511-GIIi and pSEVA6511-GIIi (Km) for Ll.LtrB/LtrA transcription as compared to the earlier constructs transcribed with the more processive T7 RNAP.

In order to move the system on to *P. putida*, the retargeting region of the intron was modified for insertion into the same previously selected locus of the *pyrF* gene, thereby generating pSEVA6511-GIIi-pyrF and pSEVA6511-GIIi (Km)-pyrF. Again, we were unable to find retrohomed mutants based on RAM. Nonetheless, empty Ll.LtrB was able to insert into *pyrF,* which verified the performance of pSEVA6511-GIIi-pyrF in this microorganism despite the change of the vector system (Figure below D-F). With this plasmid, we observed an increase in the number of $5FOA^R$ CFU of ≥ 10-fold relative to the level of spontaneous mutations (Figure below D), e.g. we found 3 colonies out of 16, which gave a frequency ~ 18 % (Figure below F, top gel). The ratio of $5FOA^R$ Ura^S was determined by patching colonies on plates with and without uracil as explained in the main text. The final frequency of authentic *pyrF* mutants created by intron insertion was calculated on the basis of colonies tested with PCR for the presence of Ll.LtrB (E). Consistent with earlier

data, the number of insertions was ~ 92.8% with no induction (a total of 13 out of 14 colonies in two replicates) and ~88.5% with induction (a total of 23 out of 26 in three replicates). This last experiment highlighted again the level of spontaneous mutations arising to 5FOA that was previously pointed out[8] and the necessity of streaking 5FOA-resistant colonies to verify their uracil auxotrophy (Figure below E) and thus the real disruption of the *pyrF* gene.

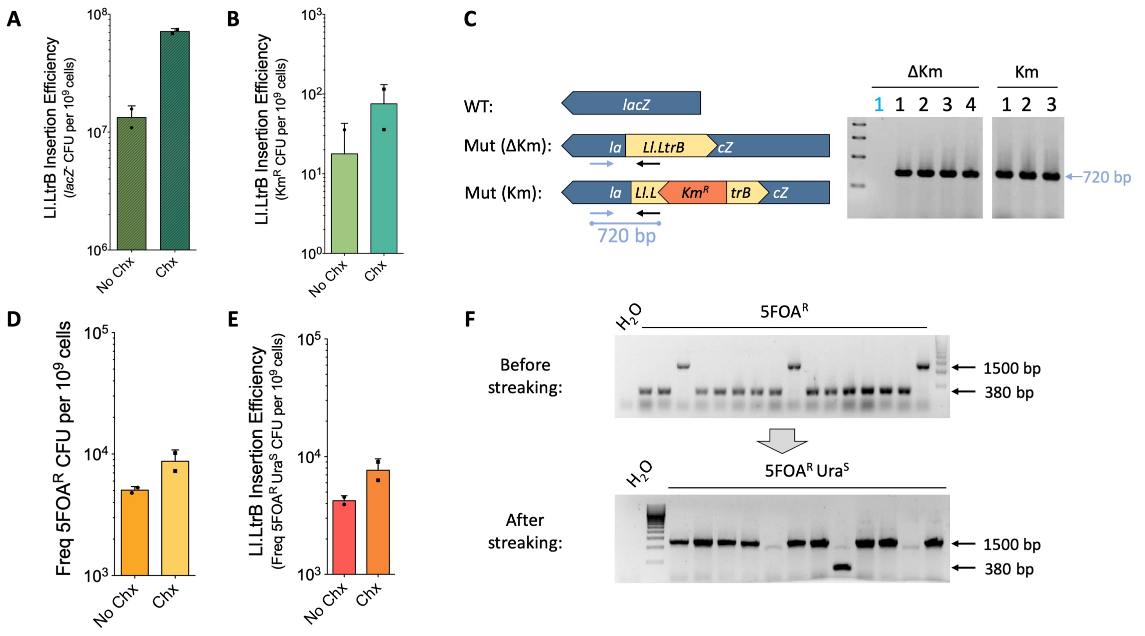

Ll.LtrB expression from engineered pSEVA6511-GIIi in *E. coli* BL21DE3 and *P. putida* KT2440. (A-C) pSEVA6511-GIIi(Ø/Km) plasmids express Ll.LtrB intron in *E. coli* BL21DE3 and inserts correctly inside *lacZ* gene. (A) Putative efficiency of insertion of Ll.LtrB from pSEVA6511-GIIi. Bar plot representing the number of white (*lacZ*-) CFU normalized to 10^9 total cells. (B) Efficiency of insertion of Ll.LtrB from pSEVA6511-GIIi(Km). The number of white, Km^R CFU was normalized to 10^9 viable cells. C) A PCR reaction where one primer anneals inside Ll.LtrB and the other anneals in the *lacZ* gene was employed in both cases to verify the correct insertion of the intron. A fragment of 720bp is generated only when Ll.LtrB is present in the correct locus. Blue numbers correspond to blue colonies and black numbers correspond to white colonies used as the template material for each reaction. (D-F) pSEVA6511-GIIi-pyrF works in *P. putida* to deliver Ll.LtrB intron into the *pyrF* gene with 5FOA counterselection. (D) Bar plot showing the frequency of $5FOA^R$ CFU normalized to 10^9 viable cells after the insertion assay. (E) Putative efficiency of insertion of Ll.LtrB with pSEVA6511-GIIi in *P. putida*. The proportion of uracil auxotrophs detected from the $5FOA^R$ population was used to determine the abundance of Ll.LtrB insertions in each population (No induction vs. induction). (F) PCR reaction using primers flanking the insertion locus inside *pyrF* gene was used with $5FOA^R$ clones (top gel) and with $5FOA^R$ and Ura^S clones (bottom gel). An amplicon of 380bp is generated if Ll.LtrB is not present (WT) while a fragment of 1500bp is amplified if the intron is present (Mut). PCR results from one replicate of the cyclohexanone-induced condition is shown. All bar graphs show the mean data of two biological replicates. WT: Wild-type, Mut: Insertion Mutant, No Chx: No cyclohexanone, Chx: 1mM cyclohexanone, $5FOA^R$: 5FOA-resistant colonies, $5FOA^R$ Ura^S: 5FOA-resistant colonies and uracil auxotrophs, H_2O: Control PCR with no template material.

SUPPLEMENTARY FIGURES

Supplementary Fig. S1. SEVA plasmids for expression of Ll.LtrB intron in a wide range of Gram-negative bacteria.

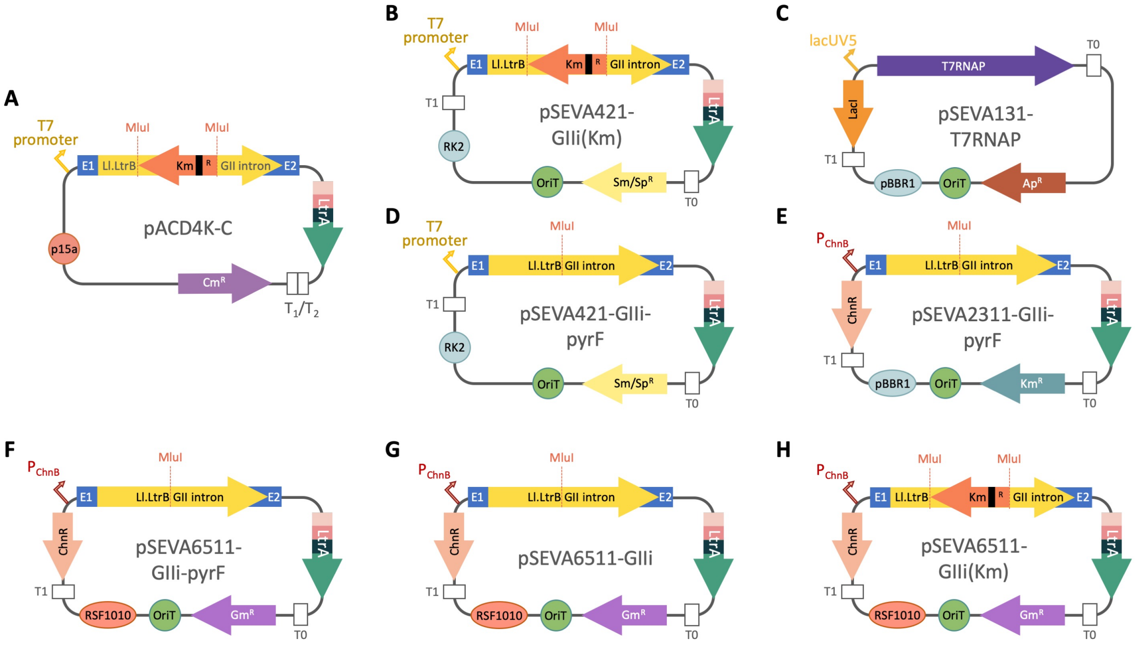

General structure and features of the main vectors used in this study. (A) Commercial plasmid pACD4K-C (Sigma-Aldrich) that was used for the amplification and subcloning of exons 1 and 2 (E1 and E2), Ll.LtrB intron and LtrA gene into the different pSEVAs generated. In this plasmid, Ll.LtrB has as cargo a Retrotransposition-Activation selectable Marker (RAM) which is composed by a kanamycin-resistance gene (Km^R) interrupted by a group I intron (black square inside the Km^R gene). Only when Ll.LtrB retrohomes and insert into DNA molecules, the group I intron is lost and the Km^R is restored. In addition, in this plasmid, Ll.LtrB is retargeted to insert into the locus 1063a of *lacZ* gene of *Escherichia coli* in antisense orientation. (B) The complete sequence including T7 promoter, E1, Ll.LtrB::Km^R, E2 and LtrA were subcloned into pSEVA421 to yield pSEVA421-GIIi(Km). This plasmid has a Sm/Sp-resistance gene, an RK2 origin of replication (low plasmid copy number) and an oriT which allows its mobilization through conjugation. Ll.LtrB in this plasmid is targeted to insert into the same locus of *lacZ* as pACD4K-C. (C) The *lac*UV5 promoter along with a short 5' region of *lacZ* gene fused to the entire T7RNAP ORF were cloned into pSEVA131, giving rise to pSEVA131-T7RNAP. This plasmid has an Ap^R gene, pBBR1 origin of replication (medium plasmid copy number) and the same oriT as the rest of pSEVAs. (D) After digesting and re-ligating pSEVA421-GIIi(Km) with MluI, the RAM was lost from Ll.LtrB, leaving an empty group II intron with no cargo sequence. Afterwards, the plasmid was retargeted to insert in antisense orientation into the 165 locus of *pyrF* gene of *Pseudomonas putida*. The resulting plasmid was named pSEVA421-GIIi-pyrF. (E) E1, Ll.LtrB (retargeted to insert into the *P. putida pyrF* gene), E2 and LtrA sequences were sub-cloned into pSEVA2311 to generate pSEVA2311-GIIi-pyrF. This plasmid has a Km^R gene, oriT, pBBR1 origin of replication and the promoter ChnR-pChnB to control the expression of Ll.LtrB and LtrA through the addition of cyclohexanone. (F) The oriV and the Km^R gene in pSEVA2311-GIIi-pyrF was substituted by an RSF1010 origin of replication and a Gm^R gene from pSEVA651, giving rise to pSEVA6511-GIIi-pyrF. (G) As in pSEVA6511-GIIi-pyrF, pSEVA6511-GIIi has an RSF1010 origin of replication and a Gm^R gene. However, the Ll.LtrB intron is retargeted to the same locus of *lacZ* gene as pSEVA421-GIIi. (H) A version of pSEVA6511-GIIi with the RAM was also generated and named as pSEVA6511-GIIi(Km). This plasmid also has a Ll.LtrB intron retargeted to insert in antisense orientation into the 1063 locus of *lacZ* gene of *E. coli.*

Supplementary Fig. S2. Preliminary approach to assess the size limit of intron-mediated delivery of DNA fragments.

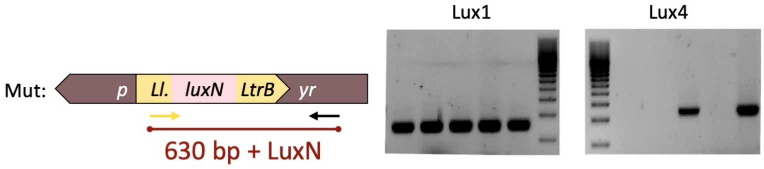

The figure to the left sketches the genomic segment (pyrF gene) where lux fragments were loaded as a cargo, next to size analysis of the PCR products of some colonies after log-phase induced cells following 5-FOA counterselection.

Supplementary Fig. S3. Total intron insertion frequency without differentiating the cargo that was being delivered.

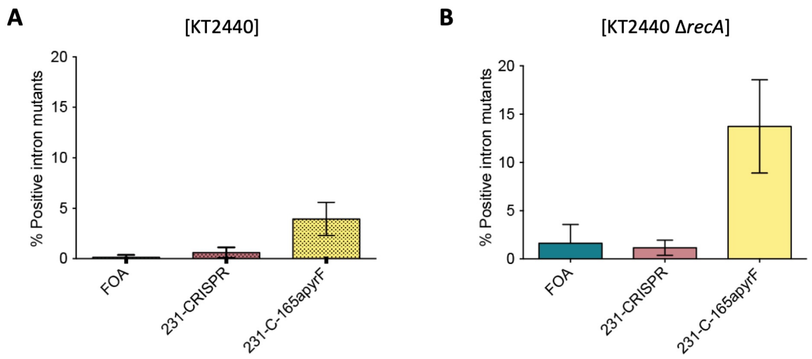

The percentages of positive clones for Ll.LtrB insertions detected with every counterselection method are plotted for (A) *P. putida* WT and (B) *P. putida* ΔrecA. The total number of positive insertions detected by PCR with each cargo were combined and plotted. The mean and standard deviation of two or three replicates are shown

Supplementary Fig. S4. Barcode generation with 3'-overlapping 119-mer oligonucleotides.

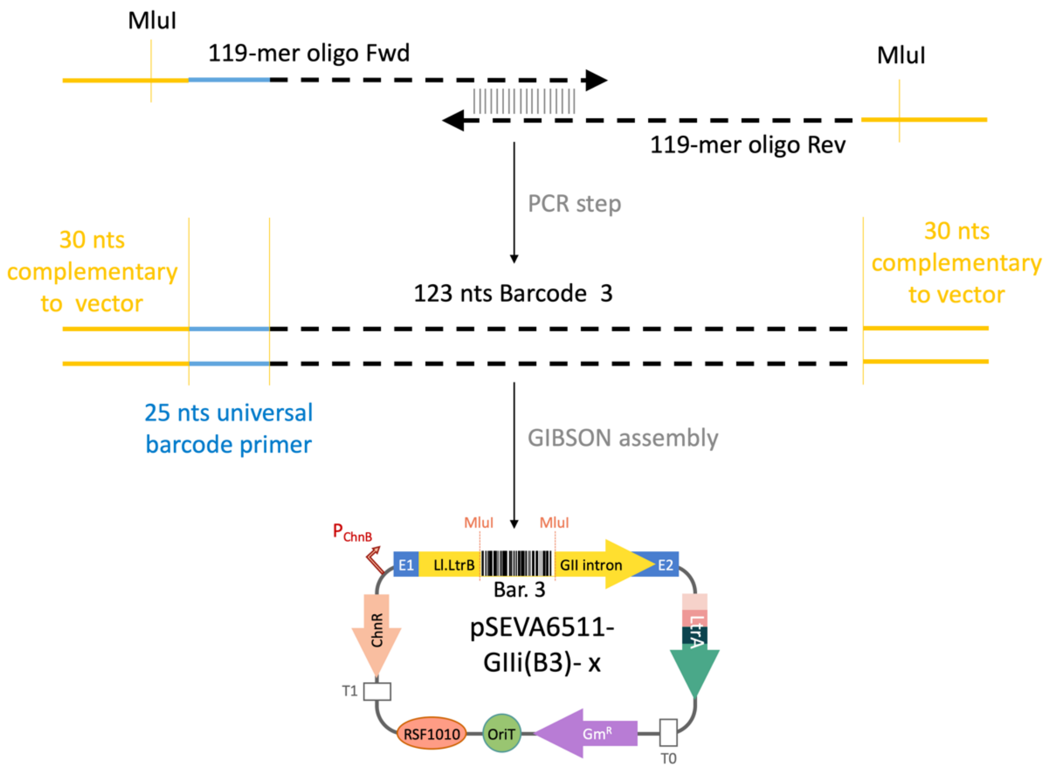

The barcode sequence retrieved from the CellRepo algorithm[9] was generated through a PCR reaction. For this, 119-mer oligonucleotides coding the barcode sequence were synthetized carrying 3' overlapping regions and 5' homologous segments with plasmid pSEVA6511-GIIi. Once generated, the PCR fragment (208 bp) was directly assembled into MluI-linearized pSEVA6511-GIIi through Gibson assembly[10,11]. Then, this plasmid was retargeted either to the Locus 1 (pSEVA6511-GIIi(B3)-37s) or to the Locus 2 (pSEVA6511-GIIi(B3)-94a) to perform the barcoding experiment. In the next figure, Locus 1 (37, 38s insertion site) and Locus 2 (94, 95a insertion site) are shown.

Supplementary Fig. S5. Application of targetrons as a barcode delivery system.

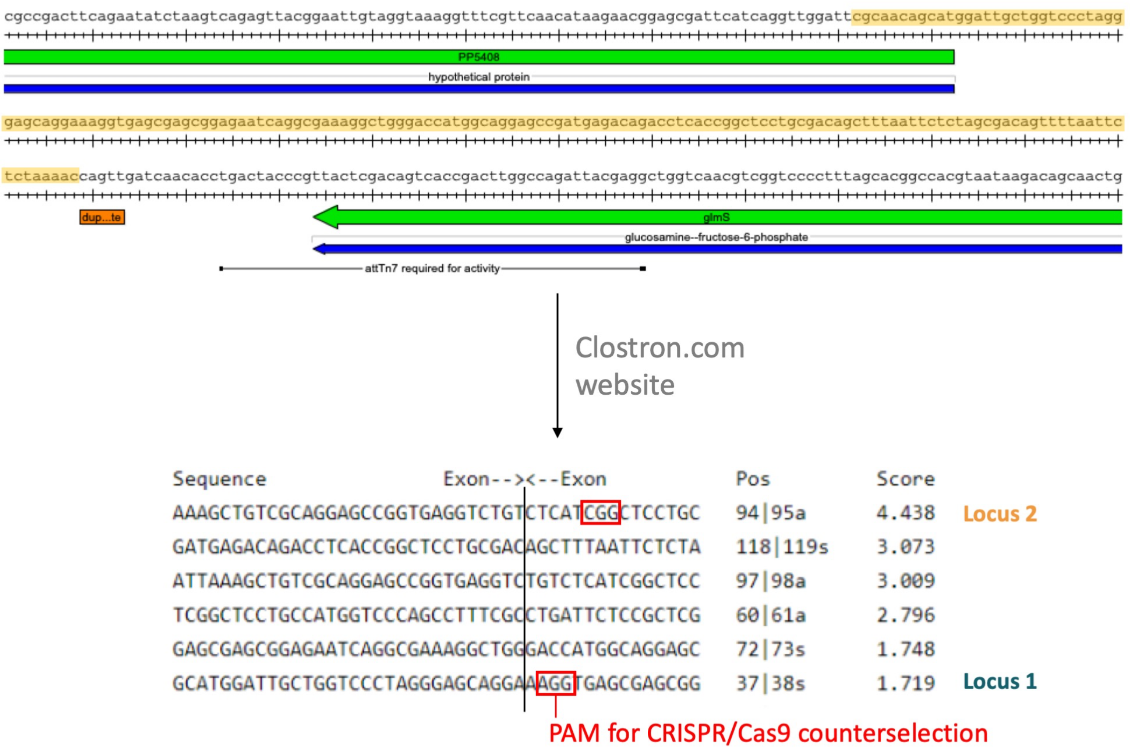

Selection of the insertion loci to be tested for the retargeting of Ll.LtrB to the close vicinity of Tn7-insertion site. Important sequences for insertion of Tn7 transposon are featured in the figure (orange box and black line). The region used in the Clostron website[12] to look for suitable insertion loci is highlighted in yellow. The retrieved list with possible retargeting loci is shown along with the two selected loci. Note the red boxes emphasizing the PAM sequences necessary for CRISPR/Cas9-mediate counterselection and used as the starting point for the design of corresponding spacers. The selected loci for retargeting are named as Locus 1 (37, 38s insertion site) and Locus 2 (94, 95a insertion site).

Supplementary Fig S6. Design of specific spacer for Locus 1 and 2 and interference experiment to test their performance for CRISPR/Cas9-mediated counterselection of Ll.LtrB::B3 group II intron.

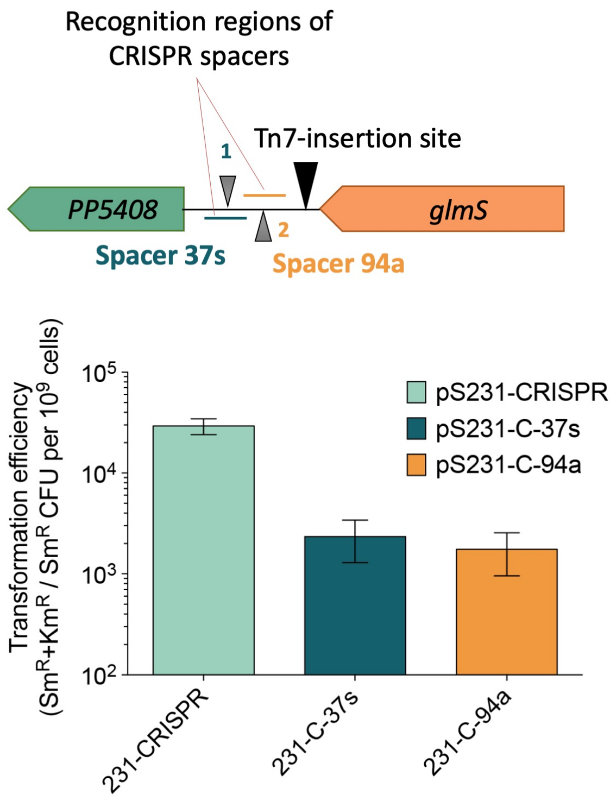

(Top) Recognition region of spacer 37s (green) and 94a (orange). Locus 1 and 2 are indicated with grey triangles. Tn7-insertion site is shown with a black triangle. (Bottom) Efficiency of cleavage of spacer 37s and 94a in comparison to control spacer present in pSEVA231-CRISPR. *P. putida* KT2440 (pSEVA421-Cas9tr) were transformed with either pSEVA231-CRISPR, pSEVA231-C-37s or pSEVA231-C-94a. Transformation efficiency was calculated by dividing the number of escapers (Sm^RKm^R) by the total number of cells (Sm^R) normalized to 10^9 cells. The mean and standard deviation of two biological replicates are represented.

Supplementary References

(1) Aparicio, T., de Lorenzo, V., and Martínez-García, E. (2018) CRISPR/Cas9-Based Counterselection Boosts Recombineering Efficiency in *Pseudomonas putida*. *Biotechnol. J. 13*, 1700161.

(2) Silva-Rocha, R., Martínez-García, E., Calles, B., Chavarría, M., Arce-Rodríguez, A., De Las Heras, A., Páez-Espino, A. D., Durante-Rodríguez, G., Kim, J., Nikel, P. I., Platero, R., and De Lorenzo, V. (2013) The Standard European Vector Architecture (SEVA): A coherent platform for the analysis and deployment of complex prokaryotic phenotypes. *Nucleic Acids Res. 41*, D666–D675.

(3) Benedetti, I., Nikel, P. I., and de Lorenzo, V. (2016) Data on the standardization of a cyclohexanone-responsive expression system for Gram-negative bacteria. *Data Br. 6*, 738–744.

(4) Martínez-García, E., Goñi-Moreno, A., Bartley, B., McLaughlin, J., Sánchez-Sampedro, L., Pascual Del Pozo, H., Prieto Hernández, C., Marletta, A. S., De Lucrezia, D., Sánchez-Fernández, G., Fraile, S., and De Lorenzo, V. (2020) SEVA 3.0: An update of the Standard European Vector Architecture for enabling portability of genetic constructs among diverse bacterial hosts. *Nucleic Acids Res. 48*, D1164–D1170.

(5) Cook, T. B., Rand, J. M., Nurani, W., Courtney, D. K., Liu, S. A., and Pfleger, B. F. (2018) Genetic tools for reliable gene expression and recombineering in *Pseudomonas putida*. *J. Ind. Microbiol. Biotechnol. 45*, 517–527.

(6) Bagdasarian, M., Lurz, R., Rückert, B., Franklin, F. C. H., Bagdasarian, M. M., Frey, J., and Timmis, K. N. (1981) Specific-purpose plasmid cloning vectors II. Broad host range, high copy number, RSF 1010-derived vectors, and a host-vector system for gene cloning in *Pseudomonas*. *Gene 16*, 237–247.

(7) Jahn, M., Vorpahl, C., Hübschmann, T., Harms, H., and Müller, S. (2016) Copy number variability of expression plasmids determined by cell sorting and Droplet Digital PCR. *Microb. Cell Fact. 15*, 211.

(8) Galvão, T. C., and De Lorenzo, V. (2005) Adaptation of the yeast *URA3* selection system to Gram-negative bacteria and generation of a *ΔbetCDE Pseudomonas putida* strain. *Appl. Environ. Microbiol. 71*, 883–892.

(9) Tellechea-Luzardo, J., Winterhalter, C., Widera, P., Kozyra, J., De Lorenzo, V., and Krasnogor, N. (2020) Linking Engineered Cells to Their Digital Twins: A Version Control System for Strain Engineering. *ACS Synth. Biol. 9*, 536–545.

(10) Gibson, D. G., Young, L., Chuang, R. Y., Venter, J. C., Hutchison, C. A., and Smith, H. O. (2009) Enzymatic assembly of DNA molecules up to several hundred kilobases. *Nat. Methods 6*, 343–345.

(11) Gibson, D. G., Glass, J. I., Lartigue, C., Noskov, V. N., Chuang, R. Y., Algire, M. A., Benders, G. A., Montague, M. G., Ma, L., Moodie, M. M., Merryman, C., Vashee, S., Krishnakumar, R., Assad-Garcia, N., Andrews-Pfannkoch, C., Denisova, E. A., Young, L., Qi, Z. N., Segall-Shapiro, T. H., Calvey, C. H., Parmar, P. P., Hutchison, C. A., Smith, H. O., and Venter, J. C. (2010) Creation of a bacterial cell controlled by a chemically synthesized genome. *Science 329*, 52–56.

(12) Heap, J. T., Pennington, O. J., Cartman, S. T., Carter, G. P., and Minton, N. P. (2007) The ClosTron: A universal gene knock-out system for the genus *Clostridium*. *J. Microbiol. Methods 70*, 452–464.
